# Transcriptomic and physiological analyses of rice seedlings under different nitrogen supplies provide insight into the regulation involved in axillary bud outgrowth

**DOI:** 10.1186/s12870-020-02409-0

**Published:** 2020-05-07

**Authors:** Rongna Wang, Junjie Qian, Zhongming Fang, Jihua Tang

**Affiliations:** 1 State Key Laboratory of Wheat and Maize Crop Science, Henan Agricultural University, Zhengzhou, 450002 China; 2 Key laboratory of Plant Resource Conservation and Germplasm Innovation in Mountainous Region (Ministry of Education), College of Agricultural Sciences, Guizhou University, Guiyang, 550025 China; 3 Guangdong Provincial Key Laboratory of Applied Botany, South China Botanical Garden, Chinese Academy of Sciences, Guangzhou, 510650 China

**Keywords:** Rice, Nitrogen, Axillary bud growth, Tiller number, Transcriptome, Cell expansion, Transcription factors, Phytohormone signalling

## Abstract

**Background:**

N is an important macronutrient required for plant development and significantly influences axillary bud outgrowth, which affects tillering and grain yield of rice. However, how different N concentrations affect axillary bud growth at the molecular and transcriptional levels remains unclear.

**Results:**

In this study, morphological changes in the axillary bud growth of rice seedlings under different N concentrations ranging from low to high levels were systematically observed. To investigate the expression of N-induced genes involved in axillary bud growth, we used RNA-seq technology to generate mRNA transcriptomic data from two tissue types, basal parts and axillary buds, of plants grown under six different N concentrations. In total, 10,221 and 12,180 DEGs induced by LN or HN supplies were identified in the basal parts and axillary buds, respectively, via comparisons to expression levels under NN level. Analysis of the coexpression modules from the DEGs of the basal parts and axillary buds revealed an abundance of related biological processes underlying the axillary bud growth of plants under N treatments. Among these processes, the activity of cell division and expansion was positively correlated with the growth rate of axillary buds of plants grown under different N supplies. Additionally, TFs and phytohormones were shown to play roles in determining the axillary bud growth of plants grown under different N concentrations. We have validated the functions of *OsGS1;2* and *OsGS2* through the rice transgenic plants with altered tiller numbers, illustrating the important valve of our transcriptomic data.

**Conclusion:**

These results indicate that different N concentrations affect the axillary bud growth rate, and our study show comprehensive expression profiles of genes that respond to different N concentrations, providing an important resource for future studies attempting to determine how axillary bud growth is controlled by different N supplies.

## Background

Rice is one of the three major crop species that provide food for more than half of the global population. Tiller number is an important agronomic trait that plays a role in rice yield by influencing the number of panicles per plant [1]. The formation of rice tillers can be divided into two developmental processes: axillary bud formation and outgrowth [2–4]. The axillary buds of tillers initiate in the axils of leaves of the basal part of shoots but then enter into dormancy. Later, these dormant axillary buds are activated and triggered by internal and environmental factors to outgrow to form tillers or branches [5]. Therefore, both axillary bud formation and outgrowth are decisive factors that affect tiller number that contribute to grain yield.

In the past few years, the regulatory mechanism of axillary bud formation and outgrowth governing the production of tillers has been studied intensively, and substantial progress has been made in the identification of key genes and important hormones involved. *MONOCULM1* (*MOC1*), *MONOCULM2* (*MOC2*), and *MONOCULM3* (*MOC3*) are required for axillary bud formation and outgrowth [3, 6, 7]. *Tillering and Dwarf1* (*TAD1*), *TEOSINTE BRANCHED1* (*OsTB1*)/*FINE CULM1* (*OsFC1*), and PCF (TCP) TF member negatively regulates rice tillering [8, 9]. In addition, LAX PANICLE1 (LAX1) and LAX2 have been reported to be regulators that control axillary bud formation [10, 11].

Plant hormone strigolactones (SLs) are well known to play negative roles in rice tillering; mutants defective in SLs biosynthesis and signalling exhibit accelerated outgrowth of tillers [12–14]. Auxin inhibits axillary bud outgrowth, and mutants defective in auxin biosynthesis and transport display accelerated outgrowth of axillary buds [15, 16]. However, cytokinin (CK) promotes the outgrowth of axillary buds, and CK-related rice mutants with increased or decreased CK contents [17–19]. In addition, mutants defective in *DWARF AND LOW*-*TILLERING* (*DLT*), whose product functions in the brassinosteroids (BRs) pathway, present relatively few tillers [20]. Recently, it is reported that BRASSINAZOLE-RESISTANT1 (BZR1) could bind to the FC1 promoter and recruit DWARF53 (D53) to inhibit its expression, suggesting that BRs play significant roles in tiller regulation [21].

Nitrogen (N) is one of the crucial macronutrients required for plants and substantially promotes the development of axillary buds of rice plants [22]. Nitrate, ammonium, and various amino acids can be utilized and assimilated by plant roots in the soil [23, 24]. Different N-transport systems exist in plants for the utilization of N, including its uptake and transport, reduction and assimilation, and translocation and remobilization. In rice, there are more than 80 *NPFs* (*NRT1/PTRs: NRT1*, *low-affinity nitrate transporter*; *PTR*, *di/tripeptide transporter*), 4 *NRT2s* and 2 *NAR2s* genes [25], and their protein functions have been extensively studied. Recently, several genes involved in nitrate or peptide transport have been reported to mediate axillary bud outgrowth and tiller number in rice [26–29]. Ammonium is assimilated into glutamine (Gln) and glutamate (Glu) by glutamine synthetase (GS) and glutamate synthase (GOGAT) [30]. In rice, there are 12 AMT (ammonium transporter) genes [31], four GS genes (*OsGS1;1*, *OsGS1;2*, *OsGS1;3*, and *OsGS2*) and two GOGAT genes (*OsNADH-GOGAT1* and *OsNADH-GOGAT1*) that participate in ammonium uptake and assimilation [32]. Amino acid permeases (AAPs) are responsible for amino acid transport, of which there are 19 members in rice [33]. Studies of *OsAAP3* [34] and *OsAAP5* [35] indicated that external N status has a profound influence on axillary bud outgrowth and tiller number in rice. A previous study revealed that the growth and yield of plants increase when nitrate and ammonium are supplied simultaneously [36], but the gene regulatory networks and signalling pathways involved in axillary bud formation and outgrowth in response to different external N supplies are still poorly understood, especially the link between N and hormones with respect to axillary bud outgrowth.

We previously reported that several genes involved in N utilization influence the tiller number and grain yield of rice [26–29, 34, 35], laying the foundation for the molecular characterization of the axillary bud growth of plants grown under different N supplies. In this study, to identify N-responsive genes associated with axillary bud growth in rice, axillary buds and basal parts at 30 days after germination under the six N concentrations were sampled for RNA sequencing (RNA-seq). By comparing with samples under normal-N (NN) concentrations, we identified 10,221 and 12,180 differentially expressed genes (DEGs), which were clustered into 9 and 10 coexpression modules respectively, based on their expression patterns at each N concentration. Genes associated with cell division and expansion, TFs, hormone transduction pathways, and other nutrient transport and signalling were associated with the axillary bud growth of plants grown under different N supplies. These results provide valuable resources and clues for studying the regulatory mechanism by which N supply affects the axillary bud growth and tiller number of rice plants.

## Results

### Growth rate of axillary buds of plants grown under different N concentrations

**Fig. 1:**
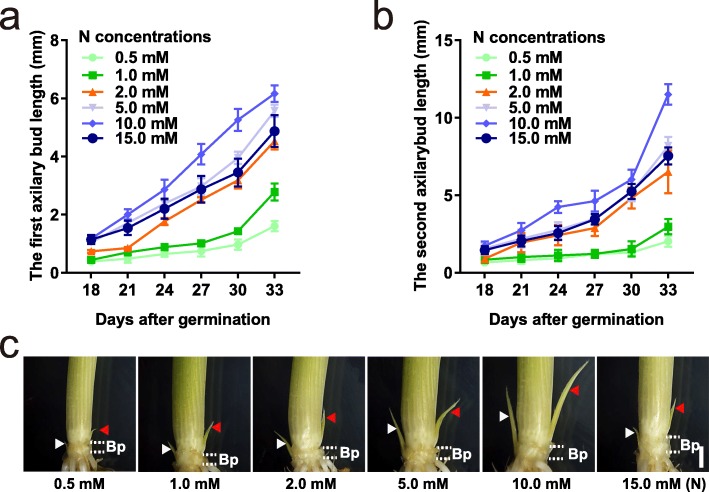
The growth of axillary bud responses to different N concentrations. **a-b** The length of the first (**b**) and second (**c**) axillary bud length under different N concentration for 18 to 33 days after germination (DAG). Data are means ± SD (*n* = 30). **c** Phenotypes of axillary bud growth under different N concentration for 30 days after germination. Scale bar, 1 mm. The axillary buds and basal parts for 30 DAG were used for transcriptional analysis. The 0.5 to 15.0 mM N concentrations were provided by 0.25–7.50 mM NH_4_NO_3_ as the nitrogen source, respectively. The parts between the white dashed lines represent the basal parts that cut for RNA-seq. The white arrowhead represents the first axillary; the red arrowhead represents the secondary axillary. Bp stands for basal part. Ab stands for axillary bud

To explore the influence of different N concentrations on axillary bud growth, rice seedlings were grown in hydroponic solutions with six concentrations of ammonium nitrate as the N source. We defined 2.0 mM N supplies by 1.0 mM ammonium nitrate as the control concentration in the standard solution for the growth rice seedlings, which was revealed in a previous report [37]. Under LN concentrations (0.5 and 1.0 mM), the lengths of the first and second axillary buds of 18- to 33-day-old seedlings were shorter than those under the optimal N concentration (Fig. 1a, b), whereas when the seedlings were grown under 5.0 mM and 10.0 mM N concentrations, the lengths of the first and second axillary buds were longer. Remarkably, when the N concentration reached 15.0 mM, the rice seedlings presented axillary buds that were shorter than the seedlings grown under 5.0 mM and 10.0 mM (Fig. 1a, b). These results indicate that different N concentrations significantly influence the outgrowth of axillary buds.

### Transcriptomic profiles of basal parts and axillary buds of plants grown under different N concentrations

To investigate the regulatory mechanisms underlying the axillary bud growth of rice plants grown under different N concentrations, we collected a mixture of the first and second axillary buds and basal parts of 30-day-old rice seedlings grown under six different N concentrations for RNA-seq analysis (Fig. 1c). In total, 39.6 to 64.6 million clean reads were generated in the libraries, and more than 90% of these reads were mapped to the reference genome (Additional file 1: Table S1). The gene expression levels were estimated using the fragments per kilobase of transcript per million reads (FPKM), and the clean reads were aligned to 31,937 genes whose FPKM was greater than 1 for at least one sample, including 25,378 protein-coding genes, 5028 non-coding RNAs, and 1530 novel transcripts. The principal component analysis (PCA) results showed good consistency between two replicates (Additional file 1: Figure S1a). Remarkably, the expression profiles of the basal parts and axillary buds were highly separated by principal component 1 (PC1), and the expression profiles of the axillary buds of plants grown under different N concentrations were separated by principal component 2 (PC2). Together with the results of Pearson’s correlation coefficients (PCCs) (Additional file 1: Figure S1b), overall, the results indicated that the transcriptomic profiles are diverse enough to identify genes responsible for N effects on axillary bud growth.

**Fig. 2:**
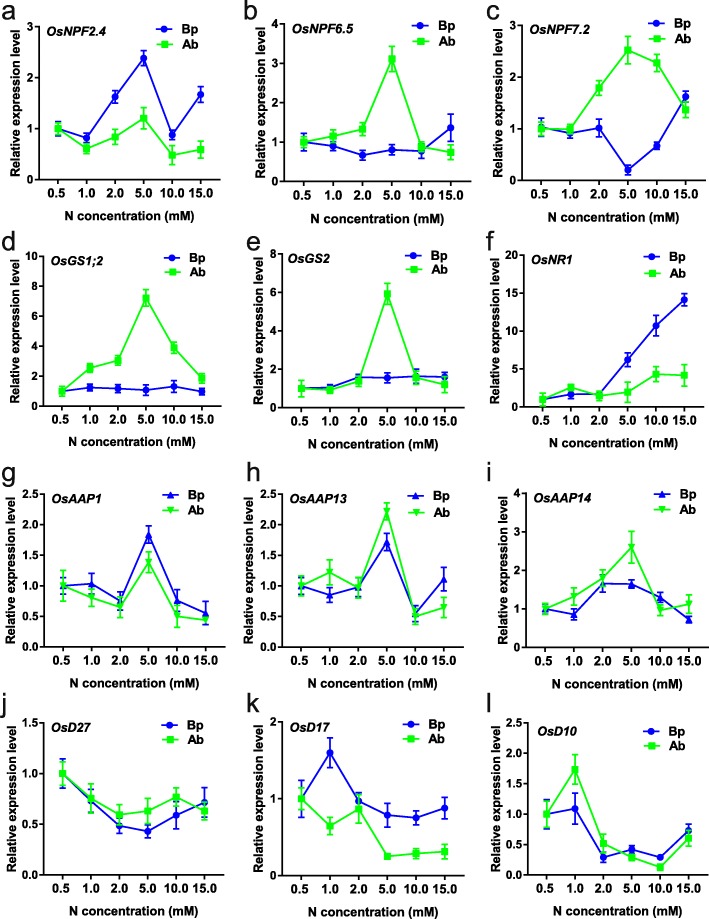
The RT-qPCR analysis of genes involved in N and SLs pathways. Ab stands for axillary bud. Bp stands for basal part. The transcript level in Bp0.5 and Ab0.5 were difined as “1” respectively. Data are means ± SD (*n* = 3)

**Fig. 3:**
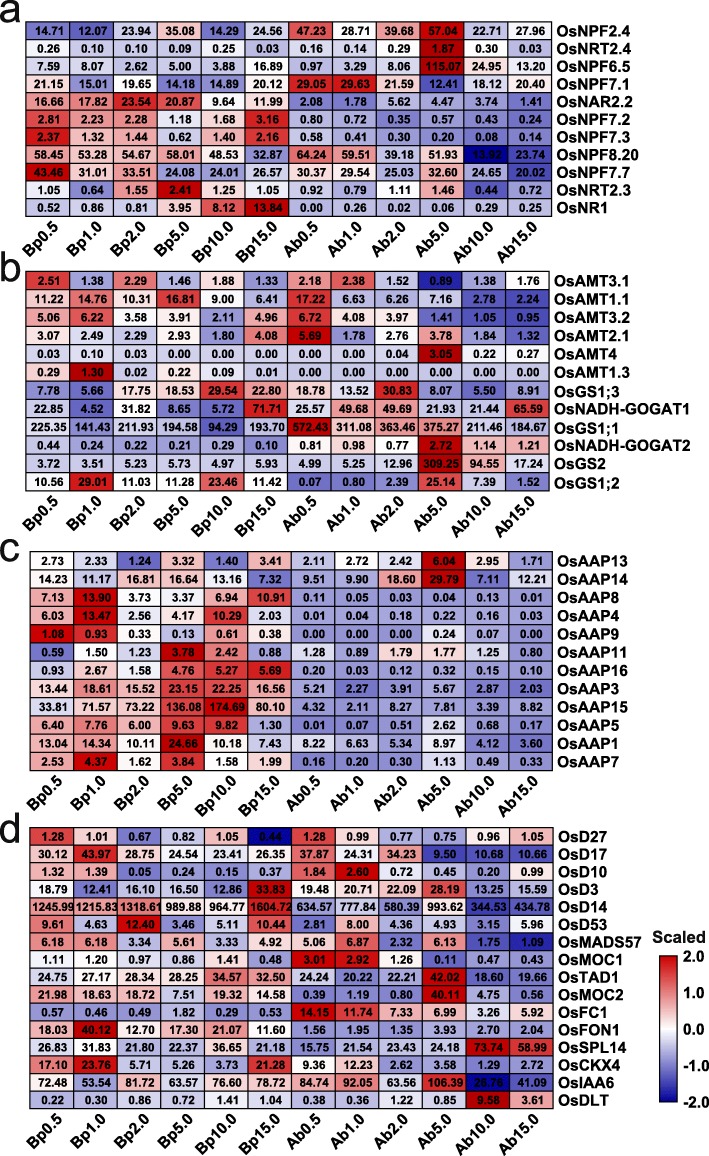
The expression of genes involved in N signaling and tiller growth under different N concentrations. Heatmaps display the expression patterns of genes involved in nitrate uptake, transport and assimilation (**a**), ammonium transporters and assimilation (**b**), amino acid transporters (**c**), and tiller bud growth (**d**) using scaled FPKM values. The numbers on the heatmaps are the FPKM values of each sample. Genes with FPKM values less than 1 in all samples were not shown. Bp stands for basal part. Ab stands for axillary bud

In addition, a total of 12 genes were randomly selected to perform the quantitative reverse transcription PCR (RT-qPCR) analysis. We found that *OsNPF6.5*, *OsNPF7.2*, *OsGS1;2*, and *OsGS2* were more highly expressed in axillary buds at 5.0 mM N concentration, *OsNPF2.4*, *OsAAP1*, *OsAAP13*, and *AAP14* were more highly expressed in both axillary buds and basal parts at 5.0 mM N concentration, suggesting that these genes might play important roles in axillary buds elongation at 5.0 mM N concentration. *OsD27*, *OsD17*, and *OsD10* were highly expressed at low N concentrations and lowly expressed at high N concentrations, which is consistent with the negative roles of SLs in axillary bud outgrowth [12–14]. In addition, the similar expression trends of these genes between the RT-qPCR results and the RNA-seq results illustrated the good quality of our transcriptomic data (Figs. 2, 3).

### Expression profiling of genes involved in N transport and axillary bud outgrowth

To investigate the genome-wide responses of genes associated with N utilization and the axillary bud growth of plants grown under different N concentrations, we analysed the expression patterns of related genes whose FPKM value was greater than 1 for at least one out of 12 samples. In both the basal parts and axillary buds, the expression levels of these genes significantly changed in response to the different N concentrations. Of the genes involved in nitrate uptake, transport and assimilation, *OsNPF7.1*, *OsNRT2.3*, *OsNPF2.4*, *OsNRF6.5*, and *OsNPF7.7* were differentially expressed in both the basal parts and axillary buds (Fig. 3a). In the basal parts, the expression of *OsNRF6.5* and *OsNPF7.1* was induced under both LN and HN conditions; however, the expression of *OsNRT2.3* and *OsNPF2.4* was induced only under HN conditions, while that of *OsNPF7.7* was induced only under LN conditions (Fig. 3a), indicating that these five genes play roles in promoting or inhibiting axillary bud growth in plants grown under different N concentrations. In addition, the expression of *OsNR1* was significantly induced in the basal parts under HN concentrations, suggesting that nitrate was assimilated under high concentrations to adapt to the high-nitrate environment. Remarkably, the expression of *OsNRF6.5*, *OsNPF7*.*1*, *OsNPF7.2*, *OsNPF7.3*, and *OsNPF7.7* was induced at a concentration 0.5 mM N (Fig. 3a), suggesting that these genes might function in activating dormant axillary buds to grow.

Of the genes involved in ammonium transporter, the expression of *OsAMT1;1*, *OsAMT2;1*, *OsAMT3;1*, and *OsAMT3;2* was induced at certain LN concentrations in both the basal parts and axillary buds (Fig. 3b), indicating that these genes play roles in ammonium uptake to meet the basic needs for rice growth. The expression of *OsAMT1;1*, *OsAMT2;1* and *OsAMT3;2* were also induced under HN conditions in the basal parts to take up additional ammonium to adapt to the high-ammonium environment. The expression of genes involved in ammonium assimilation was induced in basal parts and/or axillary buds of plants grown under certain HN concentrations. Specifically, the expression of *OsGS1;3* and *OsNADH-GOGAT1* was induced under 10.0 mM and 15.0 mM in the basal parts; the expression of *OsNADH-GOGAT2* and *OsGS2*, at both 5.0 mM and 10.0 mM in the axillary buds; and the expression of *OsGS1;2* under 10.0 mM in the basal parts and 5.0 mM in the axillary buds. These results indicate that the weakened ammonium assimilation may be a reason for the inhibited outgrowth of the axillary buds of plants grown under LN concentrations. The increased ammonium assimilation under 5.0 mM and 10.0 mM may be a reason for the accelerated outgrowth of the axillary buds, but ammonium toxicity under 15.0 mM inhibited axillary bud growth. Additionally, with the exception of that of *OsAAP13* and *OsAAP14*, the expression of most *OsAAPs* was induced in the basal parts under LN and HN conditions (Fig. 3c), especially under 5.0 and 10.0 mM N, indicating that the induction of *OsAAP* gene expression may be an adaptation to ammonium concentrations in plants and the external environment but that this induction is not the main cause of axillary bud outgrowth.

Many genes have been reported to regulate axillary bud formation and outgrowth, but whether and how these genes respond to different N concentrations are unclear. The expression of all these genes involved in axillary bud formation and outgrowth was induced at certain concentrations in the basal parts and axillary buds of rice plants (Fig. 3d). Genes involved in SL biosynthesis, including *DWARF27/17/10 (OsD27*, *OsD17*, and *OsD10)*, were highly expressed under LN concentrations in the basal parts and axillary buds, indicating that LN concentrations can promote SL biosynthesis, resulting in the suppression of axillary bud growth. However, genes involved in SL signalling, such as *DWARF3/14/53* (*OsD3*, *OsD14* and *OsD53*), were highly expressed under 15.0 mM N in the basal parts, indicating that enhanced SL signalling might play a role in ammonium toxicity to inhibit axillary bud growth. *OsMOC1* was highly expressed under 0.5 and 1.0 mM N in the axillary buds, which is consistent with its function in activating axillary meristem activity [3]. In addition, the expression of *OsCKX4* was induced at LN conditions in both the basal parts and axillary buds and under 15.0 mM N in the basal parts, indicating that the upregulation of *OsCKX4* plays a negative role in axillary bud growth, which is consistent with the results of a previous study [18]. *OsDLT*, which is regulated by the BRs pathway, coinciding with its role in branching in Arabidopsis [38], was highly expressed under 10.0 mM N in the basal parts and axillary buds. Overall, the expression profiles of these genes under different N concentrations are responsible for the N response and the growth rate of axillary buds, which is consistent with previous studies, confirming that our transcriptomic data reflect the dynamic and complex developmental process of axillary bud outgrowth of plants grown under different N concentrations.

### DEGs and their expression patterns in the basal parts and axillary buds of plants grown under different N concentrations

**Fig. 4:**
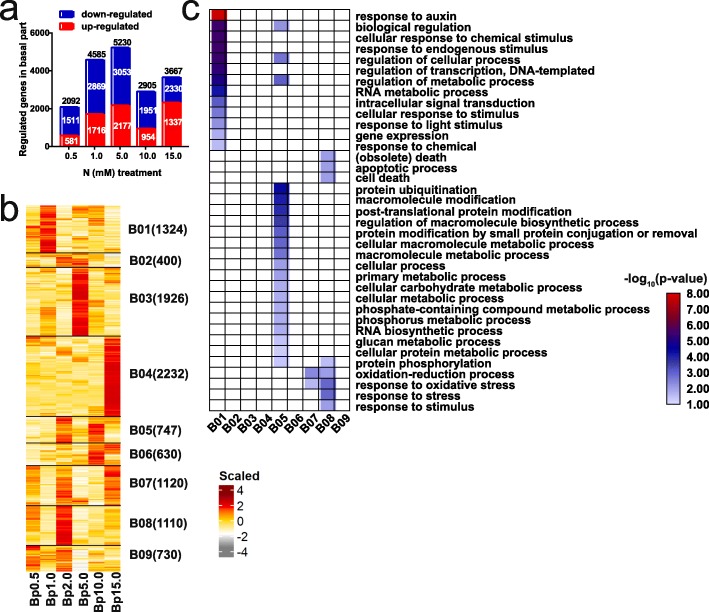
Differentially expressed genes in basal part under nitrogen concentrations. **a** The number of up and down-regulated DEGs (fold change> 2 and padj< 0.05 by DESeq2) detected between each N stress concentrations (0.5, 1.0, 5.0, 10.0, 15.0 mM) and the optimal concentration (2.0 mM) in basal part. 0.5 and 1.0 mM N are low nitrogen (LN) concentrations, while 5.0, 10.0, and 15.0 mM are high nitrogen (HN) concentrations. The number of up-, down- regulated and total DEGs were shown. **b** Clustering of the total DEGs in basal part under N stress. FPKM values were scaled per gene across basal part samples and shown as the scaled expression. Bp stands for basal part. The number of genes in each cluster is indicated beside the cluster name. **c** Enriched Gene Ontology (GO) terms within the category of biological process for the nine clusters shown in (**b**). Only significant GO terms (false discovery rate (FDR) < 0.05) are displayed

To explore the dynamic expression patterns of genes in response to different N concentrations, we identified DEGs between the optimal and each LN or HN concentration in the basal parts and axillary buds and identified the DEGs whose expression differed specifically between those two tissues at each N concentration. We found that some DEGs were specific and common between LN and HN concentrations and between both tissues (Additional file 1: Figure S2). In total, 10,221, 12,180, and 21,284 DEGs that respond to N concentrations were identified in the basal parts, in the axillary buds, and between those two tissues, respectively (Additional file 1: Table S3). We then clustered these DEGs into 9, 10, and 13 coexpression modules based on their expression patterns under the different N concentrations (Figs. 3b, 4b, Additional file 1: Figure S5b). To investigate the processes associated with N concentrations and axillary bud growth, we conducted a Gene Ontology (GO) enrichment analysis for each module (Figs. 3, 4, Additional file 1: Figures S3, S4, S5, S6).

With respect to the 10,221 DEGs in the basal parts, we found that, compared with the numbers under NN conditions, more of the DEGs were downregulated at each N concentration than that upregulated (Fig. 4a). Based on the 9 coexpression modules (B01-B09) in the basal parts, the genes could be divided into LN-responsive genes (B01), NN-responsive genes (B08), and HN-responsive genes (B03, B04, and B06). B01, B08, B03, and B04 were specific to 1.0, 2.0, 5.0, and 15.0 mM N conditions, respectively, and B06 was specific to both 10.0 mM and 15.0 mM N conditions. The LN-responsive genes in B01 were involved mainly in the response to auxin, hormone-mediated signalling pathways (auxin), cellular response to chemical stimulus/stimulus, response to endogenous stimulus/light stimulus/chemical, regulation of cellular process/transcription/metabolic process, RNA metabolic process, and gene expression. These results indicate that auxin plays important roles in the LN response to inhibit axillary bud outgrowth, which is consistent with the shortened axillary buds under LN conditions. The NN-responsive genes in B08 were enriched in the oxidation-reduction process, response to oxidative stress/stress/stimuli, apoptotic process, death, and cell death (Fig. 4c). Among them, B03 and B04 have more DEGs under 5.0 mM and 15.0 mM N concentrations respectively; however, they were not enriched in significantly biological processes at a cutoff of a false discovery rate (FDR) < 0.05 (Fig. 4c). In contrast, the expression of genes in B05 was upregulated under both 2.0 and 10.0 mM conditions, and these genes were related to protein modification, cellular macromolecule biosynthetic and metabolic processes, phosphorus (P)-containing compound metabolic process, P metabolic process, RNA biosynthetic process, and glucan metabolic process (Fig. 4c). In addition, the enriched GO terms within the molecular function category in B05 included acid amino acid ligase activity and ubiquitin-protein transferase activity (Additional file 1: Figure S3). These results indicate that the promotion of axillary bud growth of plants grown under HN conditions may be due to enhanced metabolic processes involving P and sugars, which coincides with their functions in axillary bud growth [39, 40].

**Fig. 5:**
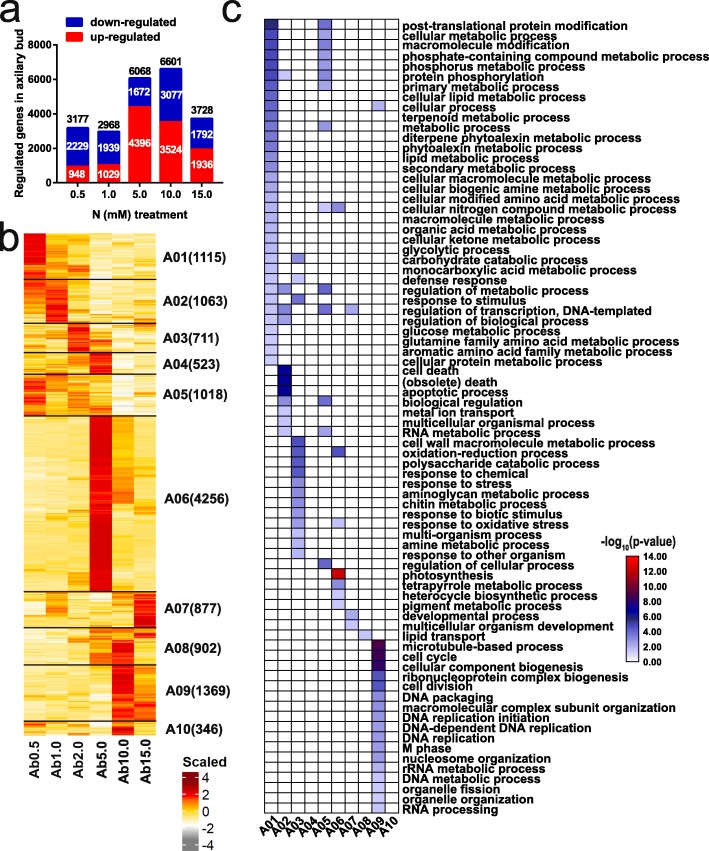
Differentially expressed genes in axillary bud under under N concentrations. **a** The number of up and down-regulated DEGs (fold change> 2 and padj< 0.05 by DESeq2) detected between each N stress concentrations (0.5, 1.0, 5.0, 10.0, 15.0 mM) and the optimal concentration (2.0 mM) in axillary bud. 0.5 and 1.0 mM N are low nitrogen (LN) concentrations, while 5.0, 10.0, and 15.0 mM are high nitrogen (HN) concentrations. The number of up-, down- regulated and total DEGs were shown. **b** Clustering of the total DEGs in axillary bud under N stress. FPKM values were scaled per gene across basal part samples and shown as the scaled expression. Ab stands for axillary bud. The number of genes in each cluster is indicated beside the cluster name. **c** Enriched Gene Ontology (GO) terms within the category of biological process for the ten clusters shown in (**b**). Only significant GO terms (false discovery rate (FDR) < 0.05) are displayed

With respect to the 12,180 DEGs in the axillary buds of plants grown under N treatments, there were more downregulated genes than upregulated genes under the 0.5 and 1.0 mM N conditions, but the opposite occurred under the 5.0, 10.0 and 15.0 mM N conditions (Fig. 5a). All 10 coexpression modules (A01-A10) under N treatments were discernibly separated according to each N concentration (Fig. 5b). The LN-responsive genes (represented by modules A01, A02, and A05) were highly expressed under the 0.5 and 1.0 mM N conditions and were related to primary and secondary metabolism, including P, lipids, terpenoids, diterpene phytoalexins, biogenic amines, amino acids, nitrogen compounds, organic acids, cellular ketones, monocarboxylic acids, and glucose, indicating that ammonium uptake and assimilation are active and mainly utilized by plants grown under LN conditions. In addition, the LN-responsive genes were also involved in protein modification, protein phosphorylation, glycolytic process, carbohydrate catabolic process, defense response, response to stimulus, apoptotic process, death, RNA metabolic process, metal ion transport. The NN-responsive genes (represented by module A03) were highly expressed under 2.0 mM N. The overrepresented terms were associated with metabolic process of various substances, including cell wall macromolecule, polysaccharide, chitin, aminoglycan, amine, and other processes, such as oxidation-reduction, response to abundant production of amino acids in the process of nitrogen metabolism. The HN-responsive genes (represented by modules A04 and A06) had more than 4 thousand DEGs that highly expressed under 5.0 mM N, were involved in photosynthesis-related processes, heterocycle biosynthetic process, cellular nitrogen compound metabolic process, oxidation-reduction process, and response to oxidative stress. Remarkably, the expression of genes involved in microtubule-based process, cell cycle, cell division, cellular component biogenesis, DNA packaging, DNA replication, nucleosome organization, M phase, DNA and RNA metabolic process, and organelle fission and organization were upregulated under 10.0 mM N in module A09, indicating that the accelerated axillary bud growth was mainly due to enhanced cell division. In addition, the expression of genes involved in the regulation of transcription, developmental processes, and multicellular organism development was upregulated under 15.0 mM N in the A07 module.

With respect to the 21,284 tissue-specific DEGs (Additional file 1: Figure S5a), 13 coexpression modules were clustered (Additional file 1: Figure S5b) and were discernibly separated into two groups: a basal tissue-specific group (BA01-BA06) and an axillary-specific group (BA07-BA13). Moreover, the axillary-specific modules (BA07-BA13) showed relatively clear expression patterns of genes in response to LN and HN conditions (Additional file 1: Figure S5b), which is consistent with the patterns of DEGs in the axillary buds (Fig. 5b). In addition, the genes enriched in various biological processes also coincided with the DEGs in the basal parts (Fig. 4c) and axillary buds (Fig. 5c); these processes include P metabolic process and response to auxin in BA04, photosynthesis-related process in BA07, and cell cycle-related processes in BA13 (Additional file 1: Figure S5c). In addition, we also provide the lists of genes and the processes they involved in that overlap and specific between the basal part and axillary bud at each N concentration (Additional file 1: Figure S7 and Table S3). Therefore, these results revealed not only the gene clusters that aggregated in certain tissues at certain N concentrations but also the underlying molecular mechanisms that regulate the responses of axillary bud growth to different N concentrations.

### Expression profiles of genes related to cell division and expansion in the basal parts and axillary buds of plants grown under different N concentrations

**Fig. 6:**
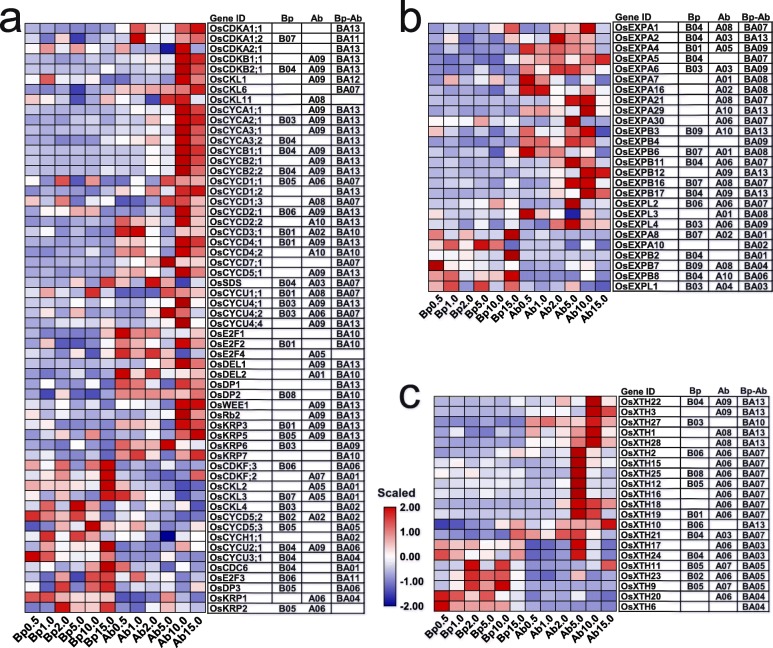
Expression profiles of genes associated with cell division (**a**) and expansion (**b** and **c**) under N concentrations. Heatmaps show the expression patterns of genes using scaled FPKM values. Genes with FPKM values less than 1 in all samples are not shown

The growth rate of the first and second axillary buds increased with increasing N concentrations ranging from 0.5 to 10.0 mM N but decreased under 15.0 mM. Based on the enriched processes associated with A09 in the axillary buds, we inferred that cell division and cell expansion are the predominant processes that determine the growth rate of the axillary buds. The expression profiles of genes involved in the cell cycle and cell division displayed tissue and N-response specificity (Fig. 6a). Most cell division-related genes were specifically expressed in the axillary buds (Fig. 6a). Remarkably, the axillary bud-specific cell division-related genes were highly expressed at HN concentrations, displaying A07, A08, A09, and A10 patterns, especially under 10.0 mM, indicating that cell division is active at HN concentrations to promote axillary bud growth.

Cell wall relaxation and loosening determine the extent of cell expansion. Similar to cell division-related genes, genes encoding cell wall relaxation- and loosening-related proteins, such as xyloglucan endotransglucosylase/hydrolase (XTH) and expansins (EXPs), exhibited tissue and N-response specificity (Fig. 6b, c) as well. Importantly, most of the *OsEXPs* were highly expressed in the axillary buds at all N concentrations except 15.0 mM, displaying A01-A06 and A08-A10 patterns (Fig. 6b). Furthermore, most of the *OsXTHs* were highly expressed in the axillary buds under 5.0 and 10.0 mM N, displaying A06 and A09 patterns (Fig. 6c). These findings indicate that cell expansion is active under 0.5 to 10.0 mM N to regulate axillary bud growth. Taken together, these results indicate that the accelerated growth of the axillary buds is made possible by cell division and expansion and that the low expression of cell expansion-related genes resulted in the suppression of the axillary bud growth of plants grown under 15.0 mM N.

### Expression dynamics of TFs in the basal parts and axillary buds of plants grown under different N concentrations

**Fig. 7:**
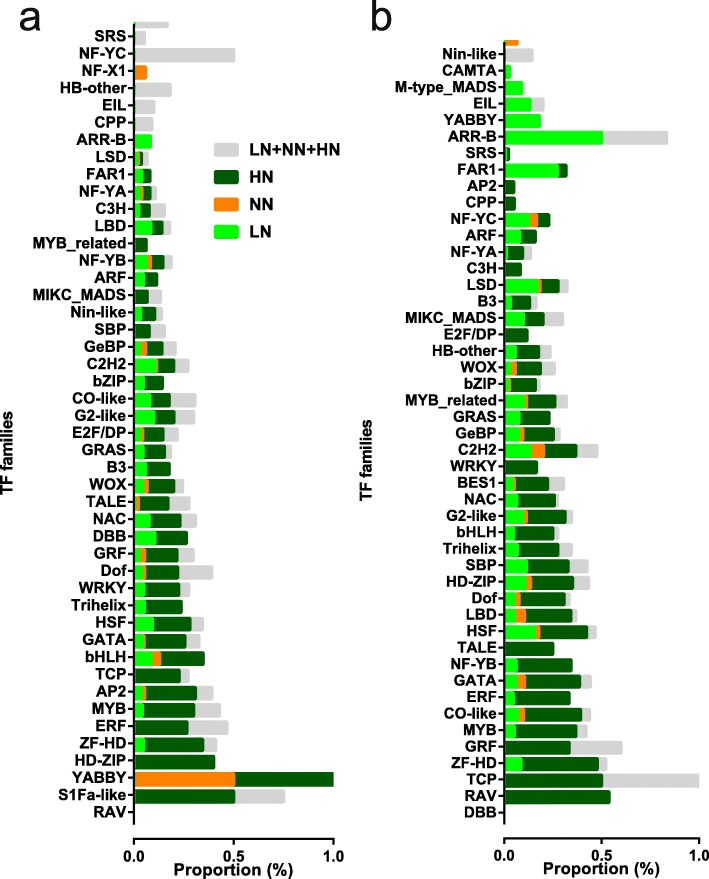
Distribution of TF families in basal part (**a**) and axilary bud (**b**) under N concentrations. LN stands for low nitrogen; NN stands for normal nitrogen, which is the control concentration in the standard solution; HN stands for high nitrogen. TF genes with FPKM values less than 1 in all samples were not shown

Because TFs are the main regulators that alter the expression of transcripts, to identify which TF families play a more important role in axillary bud growth in response to different N concentrations, we analysed the expression profiles of TF genes in detail. Of the total 1822 TF genes in rice on the Plant Transcription Factor Database (PlantTFDB), 908 TF genes within 52 families were differentially expressed; among them, 599 TF genes were classified as belonging to the B01 to B09 clusters (Additional file 1: Figure S8a), and 716 were classified as belonging to A01-A10 (Additional file 1: Figure S8b). Additionally, in the basal parts and axillary buds, more than half of these TF genes (52.1 and 55.9%, respectively) were expressed at the highest levels in response to HN stimuli, and the expression levels of only 16.2 and 22.8%, respectively, peaked in response to LN stimuli. To investigate the expression levels of members of TF families in the basal parts and axillary buds of plants grown under N stress, we calculated the total FPKM values of all TF members within a family (Additional file 1: Figure S9). We also explored the expression trends of TF families via the proportion of TF members within a family relative to the total TF members within a TF family, the information of which provided by PlantTFDB (Fig. 7).

For the basal parts, the TF gene in the LSD family was induced specifically under LN stress (Fig. 7a), which might indicate involvement in cell death under oxidative stress [41]. The expression of many TF family members was induced under HN stress, including RAV, YABBY, HOMEODOMAIN-LEUCINE ZIPPER (HD-ZIP), ZF-HD, ETHYLENE RESPONSE FACTOR (ERF), MYELOBLASTOSIS (MYB), APETALA2 (AP2), TCP, BASIC/HELIX–LOOP–HELIX (bHLH), GATA, HEAT STRESS TRANSCRIPTION FACTOR (HSF), Trihelix, DNA BINDING WITH ONE FINGER (Dof), GROWTH REGULATING FACTOR (GRF), DOUBLE B-BOX (DBB), NAM, ATAF1/2, and CUC2 (NAC), THREE-AMINO ACID-LOOP-EXTENSION (TALE), WUS HOMEOBOX-CONTAINING (WOX), B3, and GRAS family members (Fig. 7a). Together with the expression levels of TF family members (Additional file 1: Figure S9), YABBY, HD-ZIP, ZF-HD, TCP, and GRF TFs were expressed at low levels under HN conditions, whereas TALE and WOX TFs, which are involved in meristem formation or maintenance [42] and embryonic patterning [43], respectively, were moderately highly expressed under HN conditions. GATA, NAC, and B3 TFs, which are involved in auxin signalling [44] and axillary bud development [45], were highly expressed under 5.0 mM N. Trihelix TFs were highly expressed under 5.0 and 10.0 mM N. ERF, MYB, bHLH, HSF, GRAS TFs, which are involved in stress and ethylene responses [45], phosphate-starvation responses [46], and abiotic stress responses [47], were highly expressed under 10.0 and 15.0 mM N. RAV, AP2, Dof, and DBB TFs were highly expressed under 15.0 mM N, indicating their roles in inhibiting the axillary bud growth of plants grown under HN stress.

With respect to axillary buds, the expression of members of the SRS, AP2, ARR-B, B3, YABBY, and ARF TF families was induced under LN conditions (Fig. 7b). Of these TFs, the SRS and AP2 TFs were highly expressed under 0.5 mM, and the expression of the ARR-B and ARF TFs was high and low under 1.0 mM N, respectively, indicating that they play various roles in axillary buds under N stress. Members of the CO-like, GATA, NF-YB, HD-ZIP, Trihelix, NAC, BES1, GLABROUS1 ENHANCER-BINDING PROTEIN (GeBP), and CCCH ZINC FINGER (C3H) TF families were highly expressed under 5.0 mM N, whereas TCP TFs under 10.0 mM and GRF TFs were expressed at low levels under 10.0 and 15.0 mM. Among these TFs, the TCP and GRF TFs are involved in cell proliferation [48, 49], supporting a role in cell division in the axillary bud growth of plants grown under HN conditions.

### Global analysis of phytohormone signals triggered by different N concentrations

To reveal the functions of phytohormones involved in the axillary bud growth of plants grown under different N concentrations, we identified 242 DEGs associated with eight types of hormones (auxin, CK, SLs, BRs, abscisic acid (ABA), ethylene, gibberellic acid (GA), and jasmonic acid (JA); Figs. 2d, 7, Additional file 1: Figure S10). We classified the hormone-related genes into four types on the basis of their function: 1-biosynthesis, 2-degradation, 3-transport, and 4-signalling and response. The expression levels of these hormone-related genes displayed tissue- and N concentration-specificity. Additionally, more genes were highly expressed in the basal parts than in the axillary buds, suggesting that different N concentrations might regulate axillary bud growth mainly by altering hormone signals in basal parts.

**Fig. 8:**
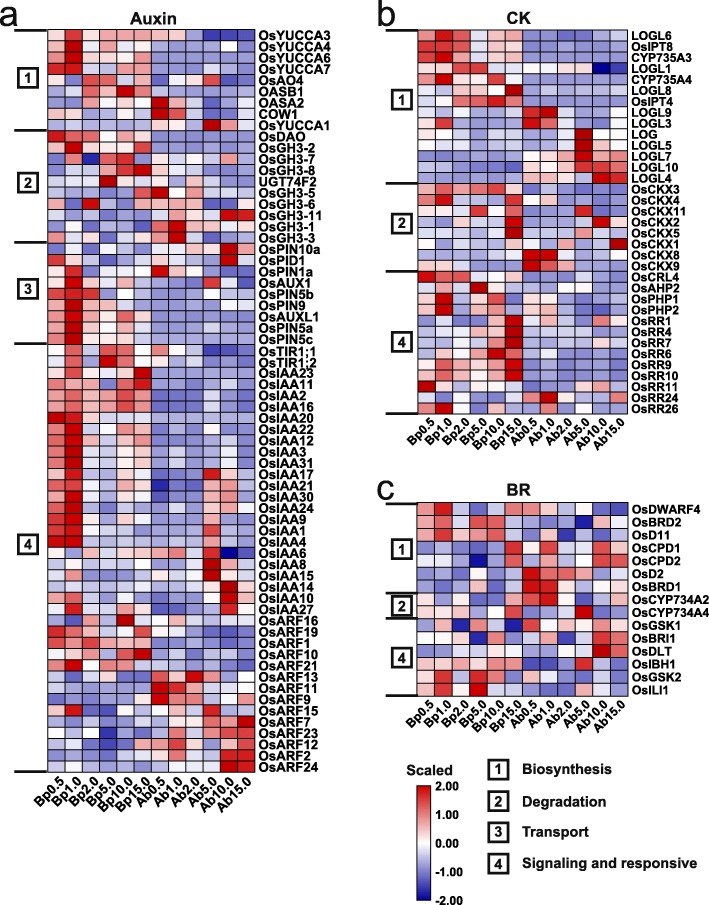
Expression profiles of genes associated with auxin (**a**), CK (**b**), and BRs (**c**) under N concentrations. Heatmaps show the expression patterns using scaled FPKM values. Genes with FPKM values less than 1 in all samples were not shown

SLs, auxin, CK, and BRs are well known to regulate axillary bud formation and outgrowth: SLs and auxin act as inhibitors, whereas CK and BRs act as promoters [21, 50–52]. Consistent with SLs as inhibitors of axillary bud growth, biosynthesis-related genes (*OsD27*, *OsD17*, and *OsD10*) were highly expressed under LN conditions, whereas signalling-related genes, such as *OsD3* and *OsD14*, were highly expressed under 15.0 mM N (Fig. 3d). For auxin, the genes related to biosynthesis and transport, such as *OsYUCCAs* and *OsPINs*, presented relatively high expression levels in the basal parts under 1.0 mM N (Fig. 8a), suggesting that enhanced auxin biosynthesis in the shoot meristem and the oscillation of the auxin gradient may be involved in axillary bud development under LN stress. These results coincide with previously reported results in which enhanced biosynthesis and transport of auxin negatively affect rice tillering [50]. Moreover, consistent with the enriched GO terms associated with B01 (Fig. 4b, c), most of the *AUX/indole-acetic acid* (*IAA*) genes were expressed at relatively high levels under 1.0 mM N, but the expression of ARFs exhibited various changes, suggesting that auxin may play complex and crucial roles in regulating the axillary bud growth of plants grown under different N concentrations. The expression of genes related to CK biosynthesis exhibited tissue specificity in the basal parts and axillary buds under both LN and HN conditions (Fig. 8b). Of which, *OsIPT4* had peak expression level at 10.0 mM N and moderate expression levels at 2.0 and 5.0 mM in basal part, and the expression of *OsCKX2* and *OsCKX4* was high under 15.0 mM N, which is consistent with CK acting as a promoter of axillary bud growth [50, 51]. Of the CK signalling- and responsive-related genes, the expression of the type A-ARRs (*OsRR1/4/7/6/9/10*) and type B-ARRs (*OsRR24/26*) was upregulated and downregulated, respectively, under 15.0 mM N in the basal parts, indicating the roles of CK signalling in inhibiting the axillary bud growth of plants grown under HN stress. For BRs, the biosynthesis and signaling related genes displayed deverse response to different N concentrations (Fig. 8c). Of these genes, *OsDLT* was reported to positively regulate rice tiller number [52], and its expression was triggered in response to 10.0 and 15.0 mM N. Recently, endogenous castasterone (CS) that the most bioactive BR in rice was reported to promote axillary bud growth under higher concentrations [21], suggesting that BRs may play positive roles in the N response to regulate axillary bud growth.

Additionally, ABA, GA, ethylene, and JA also play important roles in plant development and the abiotic stress response. Under HN conditions, ABA biosynthesis-related genes were highly expressed in the axillary buds, especially under 5.0 mM N, but they were expressed at low levels in the basal parts (Additional file 1: Figure S10a). In contrast, under HN conditions, degradation-related genes were expressed at low levels in the axillary buds but were expressed at high levels in the basal parts (Additional file 1: Figure S10a), indicating that the amount of ABA may be increased in the axillary buds under HN stress to promote their growth. ABA signalling-related genes were also highly expressed under HN stress, suggesting that ABA may preferentially function under HN stress to be involved in axillary bud growth. *OsGA20ox1*, which encodes the rate-limiting enzyme in GA biosynthesis, was expressed at high levels in the axillary buds of plants grown under LN conditions (Additional file 1: Figure S10b), whereas the expression of GA degradation-related genes (*OsGA2ox3*/4/5) was low in the axillary buds (Additional file 1: Figure S10b). These results suggest that the upregulated biosynthesis genes and downregulated degradation genes may result in increased GA levels in the axillary buds under LN conditions. The expression of the ethylene biosynthesis-related genes *ACC synthase2* (*OsACS2*) and *ACC oxidase7* (*OsACO7*) was upregulated in the axillary buds under 1.0 mM N, and the expression of *OsACS2*, *OsACO5*, and *OsACO6* was upregulated in the axillary buds under 15.0 mM N. In contrast, ethylene degradation-related genes were expressed at low levels under 15.0 mM N (Additional file 1: Figure S10c), indicating that ethylene levels might increase in axillary buds and function under 1.0 and 15.0 mM N conditions to suppress axillary bud growth. JA biosynthesis genes, such as *defective in anther dehiscence1;2* (*OsDAD1*;2), *lipoxygenases2;1* (*OsLOX2*;1), *lipoxygenases2;3* (*OsLOX2*;3), oxide synthase1 (*OsAOS1*), *OsAOS2*, allene oxide cyclase (*OsAOC*), and *jasmonate resistant1;2* (*OsJAR1*;2), were highly expressed in the basal parts under 15.0 mM N, suggesting that JA-isoleucine (JA-Ile) levels increased in the basal parts under 15.0 mM N to repress axillary bud growth.

### Dynamic expression of genes involved with potassium (K) and phosphate in plants grown under different N concentrations

Mineral nutrients are required for plant development, especially N, P, and K. To determine the interactions among N, P, and K involving axillary bud growth, we examined the expression profiles of genes associated with P and K under different N concentrations. Overall, the expression patterns showed that genes associated with P and K displayed distinct expression patterns under different N concentrations (Additional file 1: Figure S11), suggesting that different N concentrations affect the uptake and localization of P and K. The expression of the K transporter-associated *high-affinity K*^*+*^*transporters* (*OsHAK*) genes showed tissue specificity; for example, *OsHAK12/10/4/23/7/9/27* were expressed specifically in the basal parts, and *OsHAK16/17/1/19/13/22/24/18/11* were expressed specifically in the axillary buds (Additional file 1: Figure S11a). The expression of several *OsHAK*s and *OsHKT*s was induced specifically in the axillary buds under 5.0 mM N (Additional file 1: Figure S11a), indicating that increased N concentration may activate the uptake and transport of K to coordinately promote axillary bud growth. With respect to P transporters, *Pi transporter1* (*OsPT1*) was highly expressed under LN stress, increasing P uptake and remobilization. In contrast, the expression of vacuolar P transporters such as *SYG1/PHO81/XPR1-Major Facility Superfamily2* (*OsSPX-MSF2*) and *OsSPX-MSF3* displayed tissue specificity; these genes were highly expressed in the basal parts under 15.0 mM N, whereas *vacuolar processing enzyme1* (*OsVPE1*) and *OsVPE2* were highly expressed in the axillary buds under 5.0 mM N (Additional file 1: Figure S11b). The expression of most of the genes involved in P signalling and response was induced with increasing N concentration ranging from 2.0 to 10.0 mM N (Additional file 1: Figure S11c), suggesting that the increased N concentrations may activate new signalling pathways to promote axillary bud growth. In summary, increased N concentrations promote the uptake and localization of nutrients and trigger their signalling pathways to promote axillary bud growth.

### Identification of ammonium assimilation genes associate with altered tiller numbers in rice

**Fig. 9:**
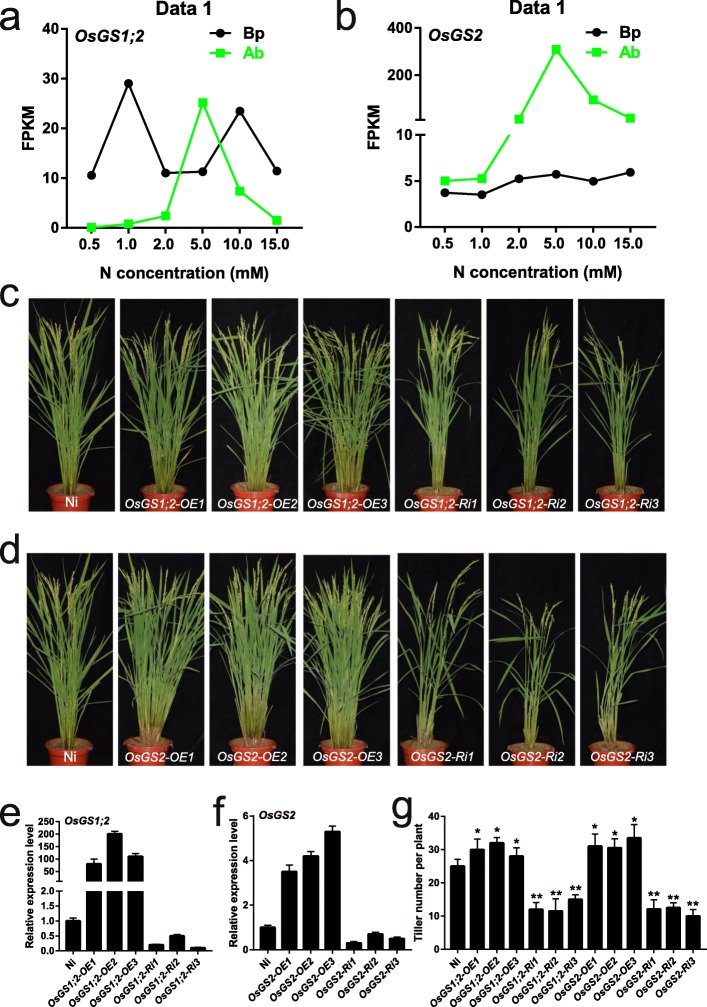
Identification and phenotypic observation of overexpression and RNAi lines of *OsGS1;2* and *OsGS2*. **a-b** Expression levels of *OsGS1;2* and *OsGS2* in basal part and axillary bud under different N concentrations. **c-d** Phenotypes of overexpression and RNAi lines of *OsGS1;2* and *OsGS2* at heading stage. Ni is the wild type (WT). OE1/2/3 represent overexpression lines. Ri1/2/3 represent RNAi lines. **e-f** The transcripts levels of *OsGS1;2* and *OsGS2* in Ni, overexpression lines, and RNAi lines. The transcript level in Ni was difined as “1”. Data are means ± SD (*n* = 3). **g** The statistic data of the tiller number at heading stage from (**e**) and (**g**). Data are means ± SD (*n* = 20)

Transcriptome analysis of basal parts and axillary buds under different N concentrations can provide important clues for the identification of novel genes that regulate axillary bud growth and tiller number in rice. In the present study, the genes *OsGS1;2* (Os03g0223400) and *OsGS2* (Os04g0659100) are involved in ammonium assimilation and were found to be highly expressed in the axillary buds under 5.0 mM N (Fig. 9a, b), indicating that they may play roles in promoting axillary bud growth and, hence, increased tiller numbers at harvest. We generated transgenic rice lines that overexpressed and downregulated the expression of *OsGS1;2* and *OsGS2*, respectively. Three overexpression (OE) lines (*OE1*, *OE2*, *OE3*) and RNA-interference (Ri) lines (*Ri1*, *Ri2*, *Ri3*) with increased and reduced expression levels of *OsGS1;2* and *OsGS2*, respectively, were selected for study (Fig. 9c-f). We observed that the tiller numbers at the heading stage of the OE lines were significantly greater than that of wild type (Fig. 9g), whereas the Ri lines had significantly fewer tillers (Fig. 9g), which coincides with the reported phenotype of the *OsGS1;2* knockout mutant [53]. Thus, these results suggest that *OsGS1;2* and *OsGS2* regulate axillary bud growth and tiller number via ammonium assimilation in rice, revealing the potential value of our transcriptomic data.

## Discussion

Axillary bud formation and outgrowth determine tiller number, panicle number, and, hence, grain yield in rice [5]. N is a crucial macronutrient for plant development. Although previous studies have elucidated the regulatory mechanisms of the axillary bud growth under N stress [22, 26, 27, 29, 35, 54], many regulators and their regulatory mechanisms are still unclear. This work revealed that the axillary bud growth rate was altered by different N concentrations, and integrated transcriptomic data from the basal parts and axillary buds of rice plants grown under different N concentrations via RNA-seq. We confirmed that N-related genes are involved in axillary bud growth and that genes controlling axillary bud growth respond to different N concentrations. The analysis identified LN- and HN-responsive genes and regulatory processes in the basal parts and axillary buds, including processes involving cell division and expansion, TFs, hormones and other nutrients. Our data reveals novel and important clues for future functional studies of axillary bud development and N sensing.

### Regulatory effects of N treatment on axillary bud growth in rice

Our study revealed that axillary bud growth is significantly influenced by different N concentrations. Under LN concentrations, axillary bud growth is inhibited. Under HN concentrations (5.0 and 10.0 mM), the growth rate increased, resulting in the production of longer axillary buds. Interestingly, possibly due to ammonium toxicity, when the N concentration increased to 15.0 mM, the axillary bud outgrowth became inhibited. Previous studies have made substantial progress in the identification of genes that respond to N [55, 56] via RNA-seq. For example, to understand early responses to ammonium in rice, genes that responded to 0, 1.0, and 10.0 mM ammonium were identified in the roots and shoots that had been treated for 4 h [55]. To investigate the expression of N-starvation-induced genes, the leaf sheaths and roots of rice plants grown under 0.072 and 1.44 mM ammonium nitrate were used to construct cDNA libraries for sequencing [56]. Together with the identification of functional genes involved in N transport, the results confirmed that N plays important roles in the regulation of axillary bud growth. Thus, these results indicate that N affects the growth rate of axillary buds but not their initiation, which is consistent with the results in a previous report [57].

### Expression patterns imply spatio-temporal specificity of genes and regulatory processes involved in the axillary bud growth of plants grown under different N concentrations

Using transcriptomic analysis, we revealed novel regulators and spatio-temporal specificity of genes and biological processes involved in the axillary bud growth of plants grown under different N concentrations. As previous studies revealed that many genes involved in the N pathway function in regulating rice axillary bud growth or tiller number [26–29, 34, 35], we first identified the potential N-transport regulators involved in the crosstalk of N and rice tillers. Among the expression of N transporter genes, *OsNPF2.4* was induced under 5.0 mM N, which is consistent with its functions in low-affinity acquisition and long-distance transport [58], supporting that *OsNPF2.4* plays a role in promoting the axillary bud growth of plants grown under HN stress. In addition, the functions of many other reported genes were confirmed in our study, such as the functions of *OsNPF7.1* [26], *OsNPF7.2* [27], and *OsNPF7.3* [29], which are differentially expressed in the basal parts or/and axillary buds in response to different N concentrations. Remarkably, many novel regulators were revealed, such as *OsNPF8.20*, *OsAMT1;1*/*3;2*/*2;1*, and *OsGS1;1*, which may suppress the axillary bud growth of plants grown under LN conditions; furthermore, *OsNRF6.5*, *OsNRT2.3*, *OsGS1;2*, *OsGS2*, and *OsAAP13*/*14* may promote the axillary bud growth of plants grown under 5.0 mM N. These results provide valuable resources for further investigation of the detailed mechanisms involved in the axillary bud growth of plants grown under N stress.

The dynamic transcriptomic data clearly demonstrated the responsive gene clusters and biological processes involved in the axillary bud growth of plants grown under various N supplies in detail. We identified tissue-specific gene clusters under different N concentrations, and more DEGs exhibited specific responses to different N concentrations in the axillary buds than in the basal parts, possibly due to the latter being the location of meristematic tissue, which is consistent with the results of expression correlations between samples (Additional file 1: Figure S1). The basal parts-specific clusters revealed that genes involved in auxin and response to external stimuli were enriched mainly under LN conditions and that P- and sugar-related processes were enriched under HN conditions, indicating that different N concentrations in the basal parts mainly trigger various stress responses, other nutrient processes and hormone signalling to affect axillary bud growth. However, the axillary bud-specific clusters under LN conditions revealed that genes involved in primary and secondary metabolism were enriched. Under HN conditions, genes involved in photosynthesis were enriched under 5.0 mM, which is consistent with the relatively high net photosynthetic rate and electron transport rate exhibited by LN-tolerant cultivars [59] and the increased division of mesophyll cells under HN fertilization [60]. Remarkably, in our data, many cell division- and expansion- related genes or/and processes were enriched under 5.0 and 10.0 mM N concentrations to promote axillary bud growth but not 15.0 mM N, suggesting the repressive functions of ammonium toxicity on cell division and elongation, as supported by previous studies [61].

### Phytohormones involved in the axillary bud growth of plants grown under different N concentrations

Phytohormones play important roles in plant development and the response to abiotic stress [62]. SLs, auxin, CK, and BRs have reported to be involved in axillary bud growth [21, 50–52]. However, it is unknown how the functions of these hormones are involved in the response to different N concentrations. In our data, under LN conditions, genes related to the biosynthesis of SLs and auxin were highly expressed, which is consistent with the roles of inhibitors of axillary bud growth [21, 50]. In addition, the upregulation of biosynthesis-related genes and the downregulation of CK degradation-related genes under HN conditions coincide with CK acting as a promoter of axillary bud growth [50, 51]. Moreover, the genes related to the signalling of these four hormones, such as *OsD3*, *OsD14*, *OsPIN1a*, *OsIAA*s, *OsIPT4*, *OsCKX*s, and *OsDLT*, were induced specifically by different N concentrations, suggesting the complex roles of hormones to affect axillary bud growth. In rice, genes involved with SLs affect N translocation to different shoot tissues [63], the free IAA content decreases under high-ammonium levels [64], and the amount of all forms of CKs increases in axillary buds and nodes in response to HN treatment [65]. In addition, the increased CS concentrations promote bud outgrowth, which is similar with SLs [21]. These results confirm that SLs, auxin, CKs, and BRs play important roles in the crosstalk between axillary bud growth and the N stress response.

In addition, genes involved in ABA biosynthesis and signalling were highly expressed in the axillary buds under HN. A previous study revealed that the signalling regulators of ABA play roles in the response to ammonium toxicity [66], indicating that ABA should be involved in the axillary bud growth of plants under different N concentrations. Luo et al. (2019) recently proposed that ABA suppresses the axillary bud growth of SL mutants and regulates SL-mediated axillary bud dormancy [67], suggesting that ABA may be involved in axillary bud dormancy to inhibit the bud outgrowth under LN conditions. Genes related to the biosynthesis and degradation of GA were expressed at high and low levels, respectively, in the axillary buds under LN conditions. Exogenous GA3 treatment inhibits axillary bud growth by altering the endogenous contents of CK, ABA, and IAA and regulating the expression of *TB1* to be expressed specifically in the axillary buds [68, 69]. These results indicate that LN stress may increase the GA content to inhibit axillary bud growth. Additionally, genes related to ethylene biosynthesis were highly expressed under 1.0 and 15.0 mM N, and ethylene inhibits cell division and DNA synthesis in the meristematic tissue of roots, shoots, and axillary buds [70], which suggesting that ethylene may repress axillary bud growth. Genes related to the biosynthesis of JA were highly expressed in the basal parts under 15.0 mM N, which may be due to the prominent role of JA in bud dormancy [71, 72], indicating that axillary buds are in a state of dormancy under 15.0 mM N.

## Conclusions

Our research provided the effects of six N concentrations on axillary bud growth to reveal that N affected axillary bud outgrowth but not its initiation, and performed detailed transcriptome analysis to provide novel regulators for future functional investigations. Based on transcriptome analysis, we conclude that the altered outgrowth of axillary bud under different N concentrations mainly due to cell division and elongation, which regulated by genes involved in stress response, N/P/sugar processes, hormone signalling, primary and secondary metabolism, and photosynthesis.

The expression levels of cell division- and expansion-related genes, TFs, hormone-related genes, and other nutrient-related genes under each N concentration in the basal parts and axillary buds provided informative clues to the regulatory mechanism and gene networks involved in the axillary bud growth of plants grown under N stress. Overall, our study provides important information concerning the axillary bud growth of plants grown under different N concentrations and reveals potential key regulators for future functional investigations.

## Methods

### Plant growth conditiesons and sample collection

The wild type rice ZH11 (*O. sativa* L. cv. *japonica*) was used in the study, which directly acquired from Prof. Mingyong Zhang, South China Botanical Garden, Chinese Academy of Sciences, that originally cultivated by Institute of Crop Sciences, Chinese Academy of Agricultural Sciences. The germinated seeds were grown in basic rice nutrient solution for 10 days. The composition of the basic solution contains 1.0 mM NH_4_NO_3_, 0.32 mM NaH_2_PO_4_, 0.51 mM K_2_SO_4_, 1.0 mM CaCl_2_, 1.65 mM MgSO_4_, 8.9 μM MnSO_4_, 0.5 μM Na_2_MoO_4_,18.4 μM H_3_BO_3_, 0.14 μM ZnSO_4_, 0.16 μM CuSO_4_ and 40.0 μM FeSO_4_. Then the seedlings were transferred and treated in basic nutrient solutions supplemented with the following concentrations of NH_4_NO_3_ as the only N source: 0.5 mM, 1.0 mM, 2.0 mM, 5.0 mM, 10.0 mM, 15.0 mM. The nutrient solutions were renewed every 3 days. The conditions in the greenhouse were 16 h (light, 32 °C)/8 h (dark, 25 °C). At 18, 21, 24, 30, 33 days after germination, the first and second axillary bud of ZH11 were measured. In addition, the axillary buds and basal parts were cut respectively for each of the six N concentrations and immediatedly frozen in liquid N for total RNA extraction. The basal parts are the position where axillary buds are born. Total RNA was extracted using TRIzol reagent (Invitrogen, Beijing, China). Two replicates of each sample were collected for RNA-sequencing.

### Library preparation for RNA sequencing and data processing

A total amount of 3 μg RNA per sample was used as input material for the RNA sample preparations. Sequencing libraries were generated using NEBNext® UltraTM RNA Library Prep Kit for Illumina® (NEB, USA) following manufacturer’s recommendations and index codes were added to attribute sequences to each sample. The clustering of the index-coded samples was performed on a cBot Cluster Generation System using TruSeq PE Cluster Kit v3-cBot-HS (Illumia) according to the manufacturer’s instructions. Using an Illumina Hiseq platform, the library preparations were sequenced and 150 bp paired-end reads were generated. Raw data (raw reads) of fastq format were firstly processed through in-house perl scripts and clean data (clean reads) were obtained by removing reads containing adapter, reads containing ploy-N and low quality reads from raw data. The clean reads were aligned to the Nipponbare reference genome (Ensemble_37) using Hisat2 v2.0.5. To estimate the expression levels of genes in samples, FPKM (fragments per kilobase of transcript per million reads) were calculated using FeatureCounts v1.5.0-p3. PCA was performed online in the website (www.omicshare.com). Expression correlations between two samples were calculated cor function in R software.

### Identification of differentially expressed genes and clustering analysis

Differential expression analysis between two samples were performed using the DESeq2 R package (1.16.1) and significantly differential expression was defined with an adjusted *P*-value < 0.05 and fold change>2. Only genes with FPKM values ≥1 in at least one sample were used to further analysis. The differentially expressed genes (DEGs) in basal part and axillary bud under different N concentrations were defined as genes significantly differential expressed between different N concentrations and the optipical concentrations. The DEGs between basal part and axillary bud were defined as genes significantly differential expressed between two tissues under each N concentration. The clustering analysis and heatmaps were performed using R packages, circlize and Complexheatmap. Other heatmaps of genes involved in certain biological processes were performed using TBtools [73].

### Functional enrichment analysis

The gene ontology (Go) enrichment analysis was performed with a cutoff of FDR ≤ 0.05 using AGRIGOv2 (http://systemsbiology.cau.edu.cn/agriGOv2/index.php), and summarized using REVIGO [74].

### Plant transformation and transgenic plant generation

To generate the overexpression vectors for *OsGS1;2* and *OsGS2*, the CDSs of *OsGS1;2* (Os03g0223400) and *OsGS2* (Os04g0659100) were cloned into the vector *pCAMBIA1306* fused with a 3 × FLAG-tag respectively. To generate the RNA interference vectors for *OsGS1;2* and *OsGS2*, the 272 bp and 217 bp fragments in coding regions of *OsGS1;2* and *OsGS2* were amplified and inserted into the *pTCK303* vector [75] respectively. Japonica seeds (Nipponbare, Ni) were acquired from Professor Yongjun Lin, College of Life Science and Technology, Huazhong Agricultural University. These vectors were then transformed into the *Agrobacterium* strain *EHA105* and introduced into the rice callus of Ni using an Agrobacterium-mediated transformation method [76]. The used primers were provided in Additional file 1: Table S2.

The T0 transgenic plants obtained were first screened by 0.1% hygromycin B (Roche Diagnostic, Rotkreuz, Switzerland). The T1 transgenic plants were screened by 0.1% hygromycin B again. Leaf samples were collected and amplified by PCR to determine the presence of DNA sequence of hygromycin B phosphotransferase using primers HYG-F and HYG-R (GenBank: E00777.1, Additional file 1: Table S2). The homozygous transgenic lines were selected based on 100% hygromycin B resistance and the presence of DNA fragment of hygromycin B. The T2 homozygous transgenic lines were used for experiments. The expression levels of *OsGS1;2* and *OsGS2* in the homozygous transgenic lines were identified by qRT-PCR.

### Real-time quantitative RT-PCR

qRT-PCR analysis were performed to detect the expression levels of genes involved in N and SLs pathways using the same samples with RNA-seq in ZH11.qRT-PCR analysis were performed to detect the expression levels of *OsGS1;2* and *OsGS2* in their overexpression and RNAi lines, and Ni. Total RNA was extracted using TRIzol reagent (Invitrogen, Beijing, China). The SYBR master mix (Invitrogen) for qRT-PCR was used. *OsActin* was used as an internal reference control. The primers were provided in Additional file 1: Table S2.

### Measurement of tiller number

For overexpression and RNAi lines of *OsGS1;2* and *OsGS2*, rice plants were grown in the paddy field from June to October at the rice experimental station of Guizhou University. At heading stage, the tiller number was measured.

## Supplementary information

**Additional file 1: Table S1.** The statistics of reads count of the RNA-seq data. **Table S2.** The primers used in our study. **Table S3.** The expression levels of all expressed genes and DEGs. **Figure S1.** Expression correlation between samples of RNA-seq. (a) Principal component analysis (PCA) shows tissue and N concentration specific samples (with repetition). Ab stands for axillary bud, Bp stands for basal part. (b) Expression correlations between two samples reveals the difference between axillary bud and basal part, and between different N concentrations. _1 and _2 stands for two repetitions. **Figure S2.** Specific and common genes between basal part and axillary bud under low N and high N conditions. (a) Venn diagram showing the overlaps among responsive genes in basal part and axillary bud under low and high N conditions. (b) The number of total and specific DEGs and TF genes in the four datasets shown in (a). (c) The common gene numbers between axillary bud and basal part under low N and high N conditions. **Figure S3.** Enriched GO terms within the category of cellular component (a) and molecular function (b) for DEGs in the ten expression patterns (B01-B09) in Fig. 4b. Only significant go terms (false discovery rate (FDR) < 0.05) are displayed. **Figure S4.** Enriched GO terms within the category of cellular component (a) and molecular function (b) for DEGs in the nine expression patterns (A01-A10) in Fig. 5b. Only significant go terms (false discovery rate (FDR) < 0.05) are displayed. **Figure S5.** Differentially expressed genes between basal part and axillary bud under each N concentration. (a) The number of up and down-regulated DEGs (fold change> 2 and padj< 0.05 by DESeq2) detected in axillary bud compared with basal part under six N concentrations. The number of up-, down- regulated and total DEGs for each N concentrations are shown. (b) Clustering the total DEGs detected between axillary bud and basal part under six N concentrations. FPKM values were scaled per gene across basal part samples and shown as the scaled expression. (c) Enriched GO terms within the category of biological process for the thirteen clusters shown in (b). Only significant go terms (false discovery rate (FDR) < 0.05) are displayed. **Figure S6.** Enriched GO terms within the category of cellular component (a) and molecular function (b) for DEGs in the thirteen expression patterns (BA01-BA13) in Additional file 1: Figure S5b. Only significant go terms (false discovery rate (FDR) < 0.05) are displayed. **Figure S7.** Overlap and specific genes between basal part and axillary bud under each N concentration. (a) Venn diagram analysis of Overlap and specific genes between basal part and axillary bud under each N concentration. (b) Enriched GO terms within the category of biological process for the overlap and specific genes (a). Only significant go terms (false discovery rate (FDR) < 0.05) are displayed. **Figure S8.** Differentially expressed TF genes in basal part (a) and axilary bud (b) under different N conditions. LN stands for low nitrogen, NN stands for normal nitrogen, HN stands for high nitrogen. B01-B09 and A01-A10 represent the clusters of DEGs in basal part (a) and axilary bud (b). Heatmaps show the expression patterns using scaled FPKM values. TF genes with FPKM values less than 1 in all 12 samples were not shown. **Figure S9.** Expression dynamics of TF families. Heatmaps show the expression patterns of TF families using scaled FPKM value of total TF genes in each family. **Figure S10.** Expression profiles of genes associated with ABA (a), GA (b), ethylene (c), and JA (d) in basal part and axillary bud under N concentrations. Heatmaps show the expression patterns using scaled FPKM values. Genes with FPKM values less than 1 in all samples were not shown. **Figure S11.** Expression profiles of genes associated with potassium transporters (a), phosphate transporters (b) and signaling (c) in basal part and axillary bud under different N concentrations. Genes with FPKM values less than 1 in all samples are not shown.

## Data Availability

The raw data collected from RNA-seq was availability in national center for biotechnology information (NCBI): https://dataview.ncbi.nlm.nih.gov/object/PRJNA627316?reviewer=doaihvgnugk1nbg6359hsnjrh7. SRA accession: PRJNA627316. The datasets used and/or analysed during the current study are available from the corresponding author on reasonable request.

## Abbreviations

AAPs: Amino acid permeasesccc
AP2: APETALA2
bHLH: BASIC/HELIX–LOOP–HELIX
BRs: Brassinosteriods
BZR1: BRASSINAZOLE-RESISTANT1
C3H: CCCH ZINC FINGER
CK: Cytokinin
D53/27/10/17/3/14: DWARF53/27/10/17/3/14
CS: Castasterone
DBB: DOUBLE B-BO
NAC: NAM, ATAF1/2, and CUC2
DEGs: Differentially expressed genes
DLT: DWARF AND LOW-TILLERING
Dof: DNA BINDING WITH ONE FINGER
ERF: ETHYLENE RESPONSE FACTOR
EXPs: Expansins
FPKM: Fragments per kilobase of transcript per million reads
GeBP: GLABROUS1 ENHANCER-BINDING PROTEIN
Gln: Glutamine
Glu: Glutamate
GO: Gene ontology
GOGAT: Glutamate synthase
GRAS: GAI, REPRESSOR OF GAI and SCR
GRF: GROWTH REGULATING FACTOR
GS: Glutamine synthetase
HD-ZIP: HOMEODOMAIN-LEUCINE ZIPPER
HN: High N
HSF: HEAT STRESS TRANSCRIPTION FACTOR
K: Potassium
LAX1/2: LAX PANICLE1/2
LN: Low N
MOC1/2/3: MONOCULM1/2/3
MYB: MYELOBLASTOSIS
N: Nitrogen
NN: Normal N
NRT1: Low-affinity nitrate transporter
OsACS2: ACC synthase2
OsACO7: ACC oxidase7
OsAOS1/2: Oxide synthase1/2
OsAOC: Allene oxide cyclase
OsCKX2/4: Cytokinin oxidase/dehydrogenase2/4
OsDAD1;2: Defective in anther dehiscence1;2
OsFC1: FINE CULM 1
OsHAK/HKT: High-affinity K+ transporters
OsJAR1;2: Jasmonate resistant1;2
OsLOX2;1/2;3: Lipoxygenases2;1/2;3
OsPT1: Pi transporter1
OsSPX-MSF2: SYG1/PHO81/XPR1-Major Facility Superfamily2
OsTB1: TEOSINTE BRANCHED 1
OsVPE1: Vacuolar processing enzyme1
P: Phosphorus
PCCs: Pearson’s correlation coefficients
PTR: Di/tripeptide transporter
RT-qPCR: Quantitative reverse transcription PCR
SLs: Strigolactones
TAD1: Tillering and Dwarf 1
TALE: THREE-AMINO ACID-LOOP-EXTENSION
TCP: TEOSINTE BRANCHED1, CYCLOIDEA, and PCF
WUS: WUSCHEL
WOX: WUSCHEL-RELATED HOMEOBOX
XTHs: Xyloglucan endotransglucosylase/hydrolase
