# Supplementary material for: Transcriptomic and physiological analyses of rice seedlings under different nitrogen supplies provide insight into the regulation involved in axillary bud outgrowth
Source: BMC Plant Biol. 2020 May 7;20:197. doi: 10.1186/s12870-020-02409-0 (PMC7206722; doi:10.1186/s12870-020-02409-0)
Supplement: Supplementary file 1 — Additional file 1: Table S1. The statistics of reads count of the RNA-seq data. Table S2. The primers used in our study. Table S3. The expression levels of all expressed genes and DEGs. Figure S1. Expression correlation between samples of RNA-seq. (a) Principal component analysis (PCA) shows tissue and N concentration specific samples (with repetition). Ab stands for axillary bud, Bp stands for basal part. (b) Expression correlations between two samples reveals the difference between axillary bud and basal part, and between different N concentrations. _1 and _2 stands for two repetitions. Figure S2. Specific and common genes between basal part and axillary bud under low N and high N conditions. (a) Venn diagram showing the overlaps among responsive genes in basal part and axillary bud under low and high N conditions. (b) The number of total and specific DEGs and TF genes in the four datasets shown in (a). (c) The common gene numbers between axillary bud and basal part under low N and high N conditions. Figure S3. Enriched GO terms within the category of cellular component (a) and molecular function (b) for DEGs in the ten expression patterns (B01-B09) in Fig. 4b. Only significant go terms (false discovery rate (FDR) < 0.05) are displayed. Figure S4. Enriched GO terms within the category of cellular component (a) and molecular function (b) for DEGs in the nine expression patterns (A01-A10) in Fig. 5b. Only significant go terms (false discovery rate (FDR) < 0.05) are displayed. Figure S5. Differentially expressed genes between basal part and axillary bud under each N concentration. (a) The number of up and down-regulated DEGs (fold change> 2 and padj< 0.05 by DESeq2) detected in axillary bud compared with basal part under six N concentrations. The number of up-, down- regulated and total DEGs for each N concentrations are shown. (b) Clustering the total DEGs detected between axillary bud and basal part under six N concentrations. FPKM values were scaled per gene acros [file 12870_2020_2409_MOESM1_ESM.zip › Additional file 1-Table S1.pdf]

Sample	Raw reads	Clean reads	Map reads	Map rate	Q20	Q30
Ab0.5_1	45404242	43838910	42109884	96.06%	98.14	94.7
Ab0.5_2	51308412	49792540	47903728	96.21%	98.19	94.81
Ab1.0_1	44298296	43547018	47497174	96.42%	98.11	94.6
Ab1.0_2	55234870	54528238	48221452	96.19%	98.12	94.61
Ab2.0_1	40418538	39653314	38231729	96.41%	98.05	94.45
Ab2.0_2	44304194	43505980	41932697	96.38%	97.79	93.86
Ab5.0_1	43105462	42392750	40933931	96.56%	98.2	94.83
Ab5.0_2	43099818	41928278	40479236	96.54%	98.24	94.98
Ab10.0_1	50193446	49258958	42059536	96.58%	98.06	94.5
Ab10.0_2	51958484	50131148	52766658	96.77%	98.14	94.72
Ab15.0_1	48456034	47004968	45366070	96.51%	98.04	94.49
Ab15.0_2	47872540	46277946	44623021	96.42%	98.08	94.58
Bp0.5_1	49523606	47838764	45919948	95.99%	97.54	93.01
Bp0.5_2	49282180	48531896	46816841	96.47%	98.06	94.43
Bp1.0_1	45546382	44270510	44034311	95.82%	98.05	94.52
Bp1.0_2	45365598	43710894	62277554	96.31%	98.11	94.6
Bp2.0_1	55050216	53887042	51935890	96.38%	97.93	94.15
Bp2.0_2	42244552	41574742	40287842	96.90%	98.29	95
Bp5.0_1	52868136	51332238	48752360	94.97%	98.35	95.15
Bp5.0_2	47550158	45810682	43618616	95.21%	98.23	94.85
Bp10.0_1	46736506	45954070	42095532	95.09%	98.22	94.87
Bp10.0_2	65766530	64660648	41732861	95.47%	98.13	94.63
Bp15.0_1	55478002	54373684	52448001	96.46%	98.25	94.98
Bp15.0_2	48938740	46892794	44977394	95.92%	98.22	94.95
