# Supplementary material for: Transcriptomic and physiological analyses of rice seedlings under different nitrogen supplies provide insight into the regulation involved in axillary bud outgrowth
Source: BMC Plant Biol. 2020 May 7;20:197. doi: 10.1186/s12870-020-02409-0 (PMC7206722; doi:10.1186/s12870-020-02409-0)
Supplement: Supplementary file 1 — Additional file 1: Table S1. The statistics of reads count of the RNA-seq data. Table S2. The primers used in our study. Table S3. The expression levels of all expressed genes and DEGs. Figure S1. Expression correlation between samples of RNA-seq. (a) Principal component analysis (PCA) shows tissue and N concentration specific samples (with repetition). Ab stands for axillary bud, Bp stands for basal part. (b) Expression correlations between two samples reveals the difference between axillary bud and basal part, and between different N concentrations. _1 and _2 stands for two repetitions. Figure S2. Specific and common genes between basal part and axillary bud under low N and high N conditions. (a) Venn diagram showing the overlaps among responsive genes in basal part and axillary bud under low and high N conditions. (b) The number of total and specific DEGs and TF genes in the four datasets shown in (a). (c) The common gene numbers between axillary bud and basal part under low N and high N conditions. Figure S3. Enriched GO terms within the category of cellular component (a) and molecular function (b) for DEGs in the ten expression patterns (B01-B09) in Fig. 4b. Only significant go terms (false discovery rate (FDR) < 0.05) are displayed. Figure S4. Enriched GO terms within the category of cellular component (a) and molecular function (b) for DEGs in the nine expression patterns (A01-A10) in Fig. 5b. Only significant go terms (false discovery rate (FDR) < 0.05) are displayed. Figure S5. Differentially expressed genes between basal part and axillary bud under each N concentration. (a) The number of up and down-regulated DEGs (fold change> 2 and padj< 0.05 by DESeq2) detected in axillary bud compared with basal part under six N concentrations. The number of up-, down- regulated and total DEGs for each N concentrations are shown. (b) Clustering the total DEGs detected between axillary bud and basal part under six N concentrations. FPKM values were scaled per gene acros [file 12870_2020_2409_MOESM1_ESM.zip › Additional file1-Figure S4.pdf]

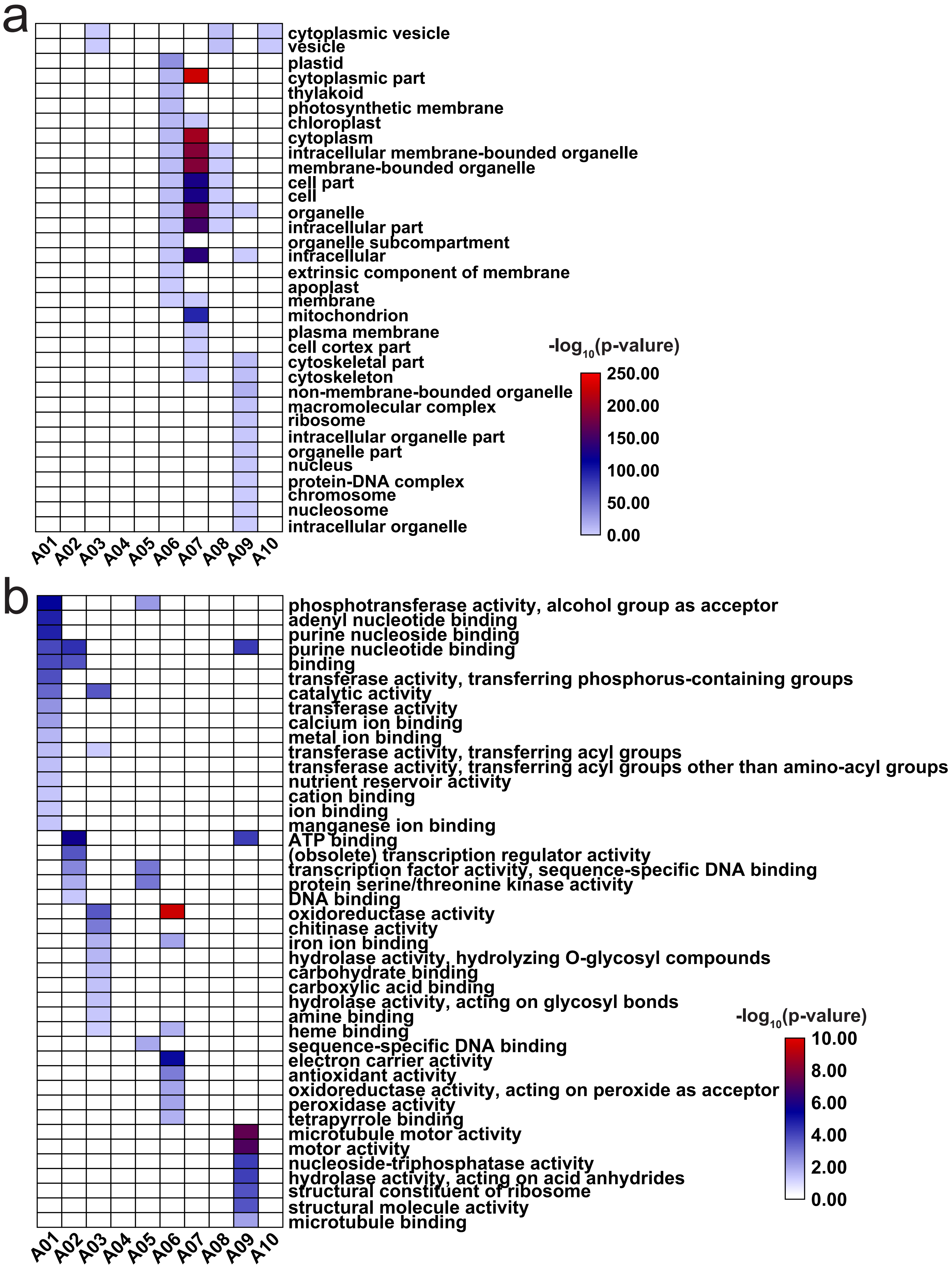

a
cytoplasmic vesicle
vesicle
plastid
cytoplasmic part
thylakoid
photosynthetic membrane
chloroplast
cytoplasm
intracellular membrane-bounded organelle
membrane-bounded organelle
cell part
cell
organelle
intracellular part
organelle subcompartment
intracellular
extrinsic component of membrane
apoplast
membrane
mitochondrion
plasma membrane
cell cortex part
cytoskeletal part
cytoskeleton
non-membrane-bounded organelle
macromolecular complex
ribosome
intracellular organelle part
organelle part
nucleus
protein-DNA complex
chromosome
nucleosome
intracellular organelle
-log10(p-valure)
250.00
200.00
150.00
100.00
50.00
0.00
A01 A02 A03 A04 A05 A06 A07 A08 A09 A10
b
phosphotransferase activity, alcohol group as acceptor
adenyl nucleotide binding
purine nucleoside binding
purine nucleotide binding
binding
transferase activity, transferring phosphorus-containing groups
catalytic activity
transferase activity
calcium ion binding
metal ion binding
transferase activity, transferring acyl groups
transferase activity, transferring acyl groups other than amino-acyl groups
nutrient reservoir activity
cation binding
ion binding
manganese ion binding
ATP binding
(obsolete) transcription regulator activity
transcription factor activity, sequence-specific DNA binding
protein serine/threonine kinase activity
DNA binding
oxidoreductase activity
chitinase activity
iron ion binding
hydrolase activity, hydrolyzing O-glycosyl compounds
carbohydrate binding
carboxylic acid binding
hydrolase activity, acting on glycosyl bonds
amine binding
heme binding
sequence-specific DNA binding
electron carrier activity
antioxidant activity
oxidoreductase activity, acting on peroxide as acceptor
peroxidase activity
tetrapyrrole binding
microtubule motor activity
motor activity
nucleoside-triphosphatase activity
hydrolase activity, acting on acid anhydrides
structural constituent of ribosome
structural molecule activity
microtubule binding
-log10(p-valure)
10.00
8.00
6.00
4.00
2.00
0.00
A01 A02 A03 A04 A05 A06 A07 A08 A09 A10
