# Supplementary figures and images for: Transcriptomic and physiological analyses of rice seedlings under different nitrogen supplies provide insight into the regulation involved in axillary bud outgrowth
Source: BMC Plant Biol. 2020 May 7;20:197. doi: 10.1186/s12870-020-02409-0 (PMC7206722; doi:10.1186/s12870-020-02409-0)

a

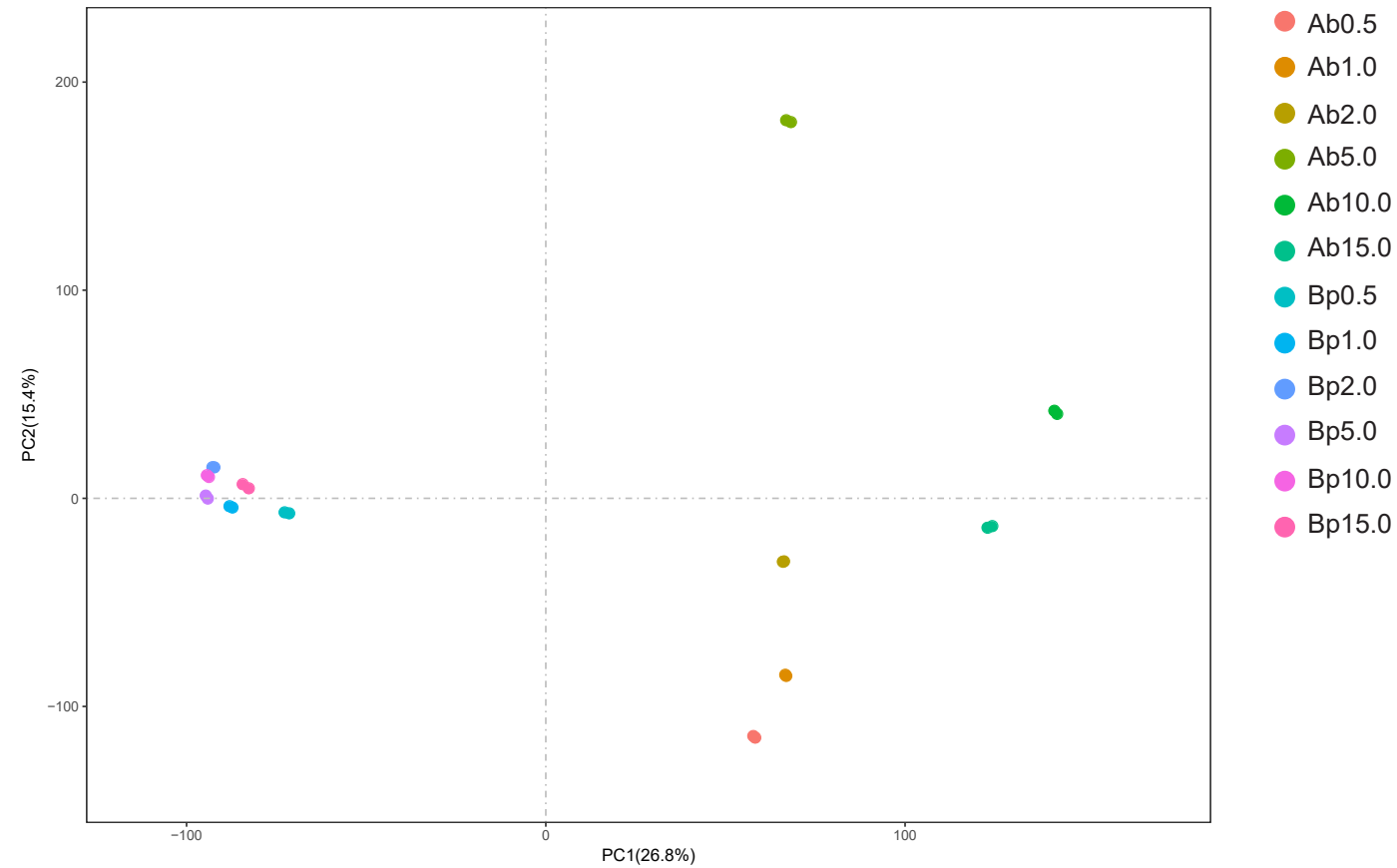

b

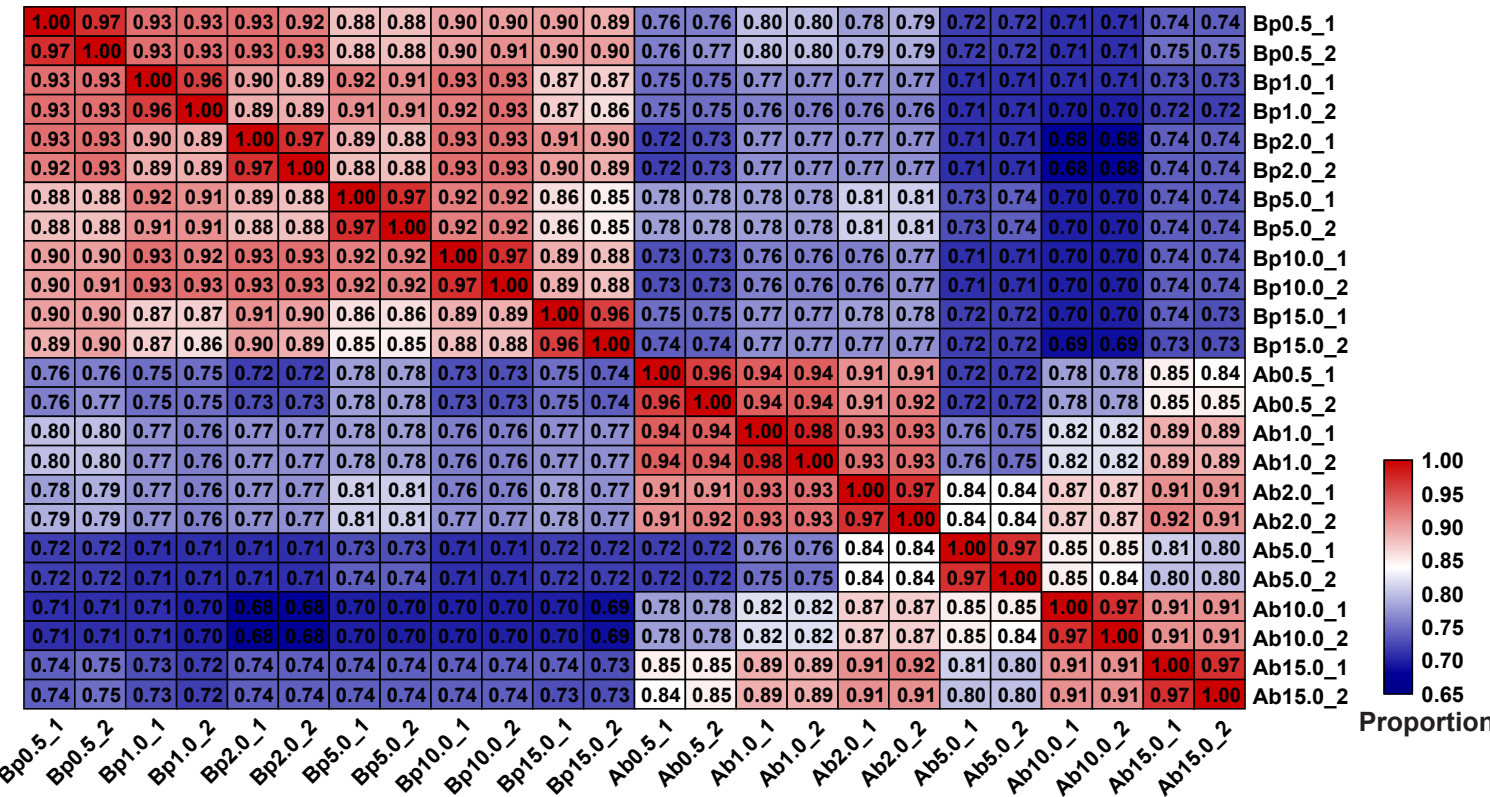

Supplement: Supplementary file 1 — Additional file 1: Table S1. The statistics of reads count of the RNA-seq data. Table S2. The primers used in our study. Table S3. The expression levels of all expressed genes and DEGs. Figure S1. Expression correlation between samples of RNA-seq. (a) Principal component analysis (PCA) shows tissue and N concentration specific samples (with repetition). Ab stands for axillary bud, Bp stands for basal part. (b) Expression correlations between two samples reveals the difference between axillary bud and basal part, and between different N concentrations. _1 and _2 stands for two repetitions. Figure S2. Specific and common genes between basal part and axillary bud under low N and high N conditions. (a) Venn diagram showing the overlaps among responsive genes in basal part and axillary bud under low and high N conditions. (b) The number of total and specific DEGs and TF genes in the four datasets shown in (a). (c) The common gene numbers between axillary bud and basal part under low N and high N conditions. Figure S3. Enriched GO terms within the category of cellular component (a) and molecular function (b) for DEGs in the ten expression patterns (B01-B09) in Fig. 4b. Only significant go terms (false discovery rate (FDR) < 0.05) are displayed. Figure S4. Enriched GO terms within the category of cellular component (a) and molecular function (b) for DEGs in the nine expression patterns (A01-A10) in Fig. 5b. Only significant go terms (false discovery rate (FDR) < 0.05) are displayed. Figure S5. Differentially expressed genes between basal part and axillary bud under each N concentration. (a) The number of up and down-regulated DEGs (fold change> 2 and padj< 0.05 by DESeq2) detected in axillary bud compared with basal part under six N concentrations. The number of up-, down- regulated and total DEGs for each N concentrations are shown. (b) Clustering the total DEGs detected between axillary bud and basal part under six N concentrations. FPKM values were scaled per gene acros [file 12870_2020_2409_MOESM1_ESM.zip › Additional file1-Figure S1.pdf]

a

ABA

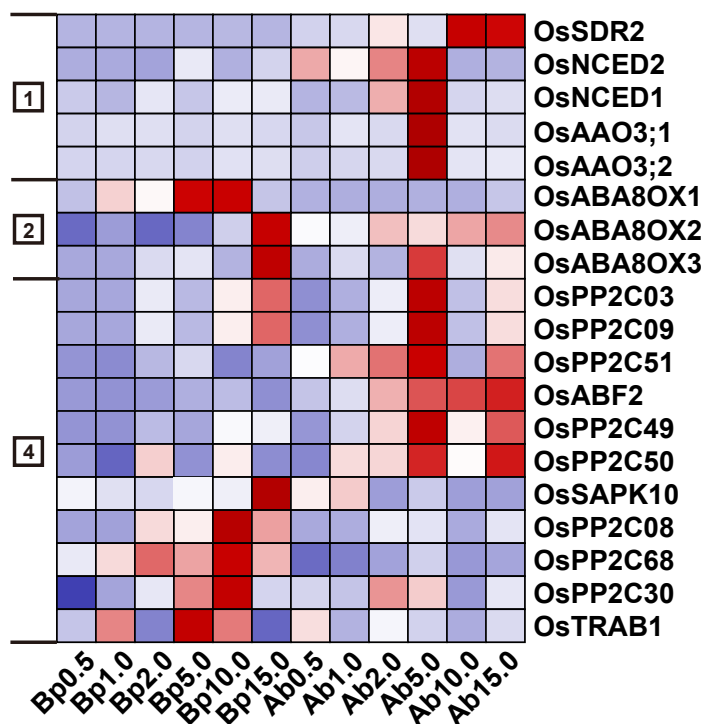

b

GA

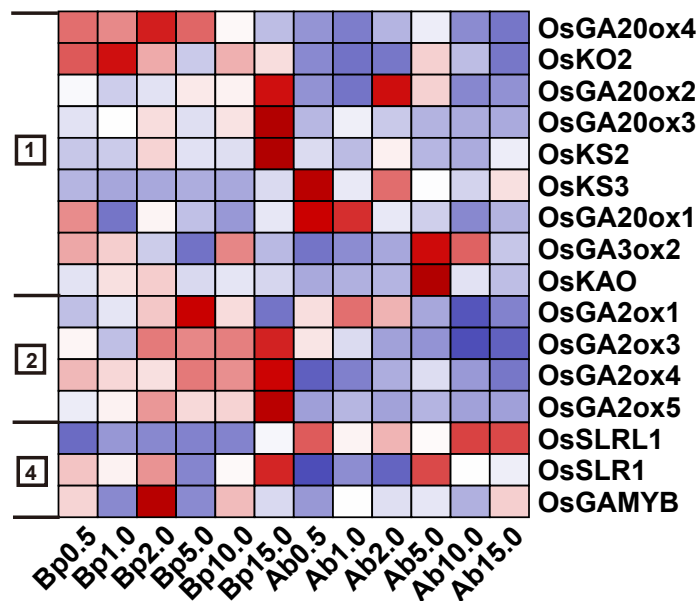

c

Ethylene

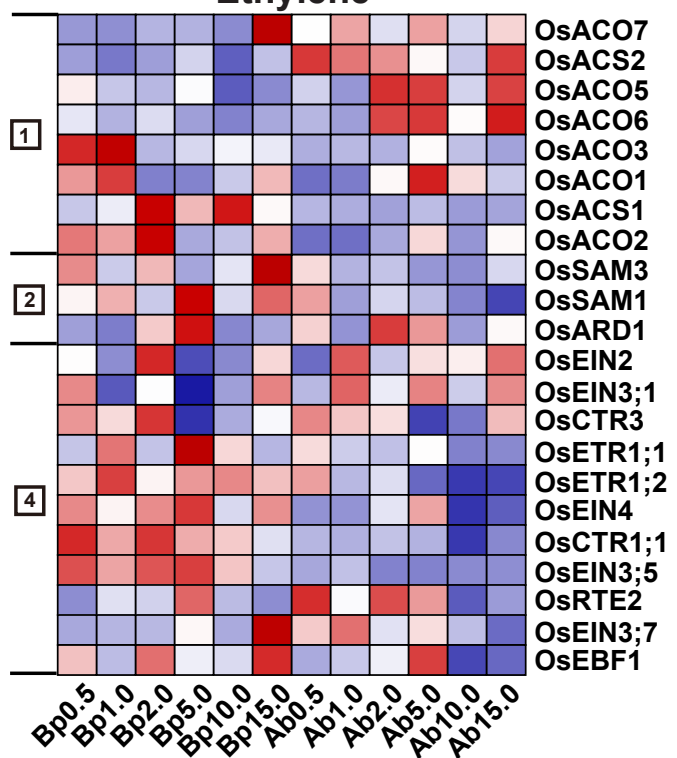

Scaled

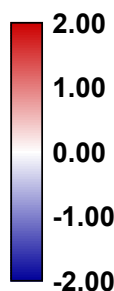

- 1 Biosynthesis
- 2 Degradation
- 3 Transport
- 4 Signaling and responsive

d

JA

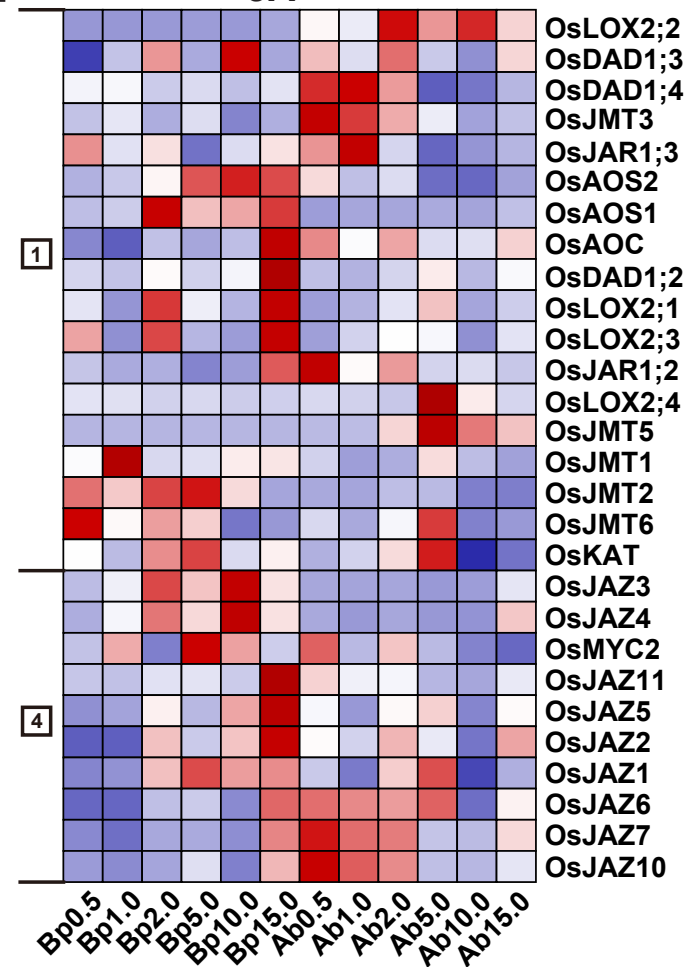

Supplement: Supplementary file 1 — Additional file 1: Table S1. The statistics of reads count of the RNA-seq data. Table S2. The primers used in our study. Table S3. The expression levels of all expressed genes and DEGs. Figure S1. Expression correlation between samples of RNA-seq. (a) Principal component analysis (PCA) shows tissue and N concentration specific samples (with repetition). Ab stands for axillary bud, Bp stands for basal part. (b) Expression correlations between two samples reveals the difference between axillary bud and basal part, and between different N concentrations. _1 and _2 stands for two repetitions. Figure S2. Specific and common genes between basal part and axillary bud under low N and high N conditions. (a) Venn diagram showing the overlaps among responsive genes in basal part and axillary bud under low and high N conditions. (b) The number of total and specific DEGs and TF genes in the four datasets shown in (a). (c) The common gene numbers between axillary bud and basal part under low N and high N conditions. Figure S3. Enriched GO terms within the category of cellular component (a) and molecular function (b) for DEGs in the ten expression patterns (B01-B09) in Fig. 4b. Only significant go terms (false discovery rate (FDR) < 0.05) are displayed. Figure S4. Enriched GO terms within the category of cellular component (a) and molecular function (b) for DEGs in the nine expression patterns (A01-A10) in Fig. 5b. Only significant go terms (false discovery rate (FDR) < 0.05) are displayed. Figure S5. Differentially expressed genes between basal part and axillary bud under each N concentration. (a) The number of up and down-regulated DEGs (fold change> 2 and padj< 0.05 by DESeq2) detected in axillary bud compared with basal part under six N concentrations. The number of up-, down- regulated and total DEGs for each N concentrations are shown. (b) Clustering the total DEGs detected between axillary bud and basal part under six N concentrations. FPKM values were scaled per gene acros [file 12870_2020_2409_MOESM1_ESM.zip › Additional file1-Figure S10.pdf]

a

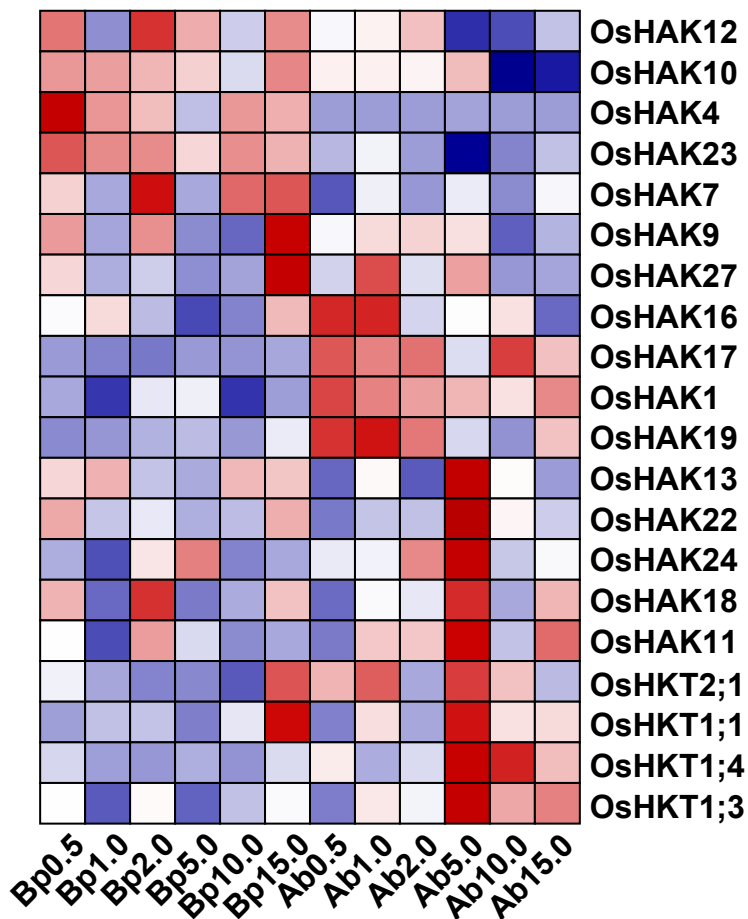

b

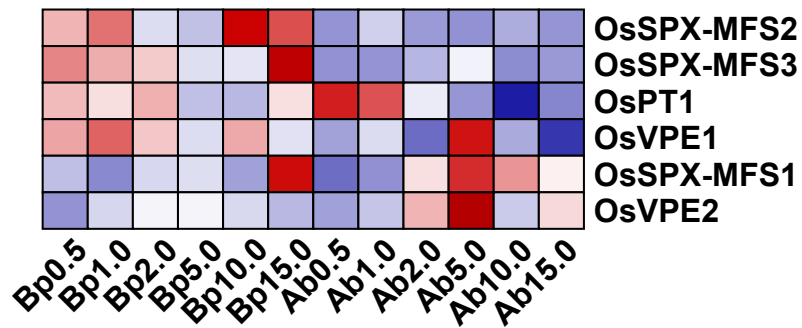

c

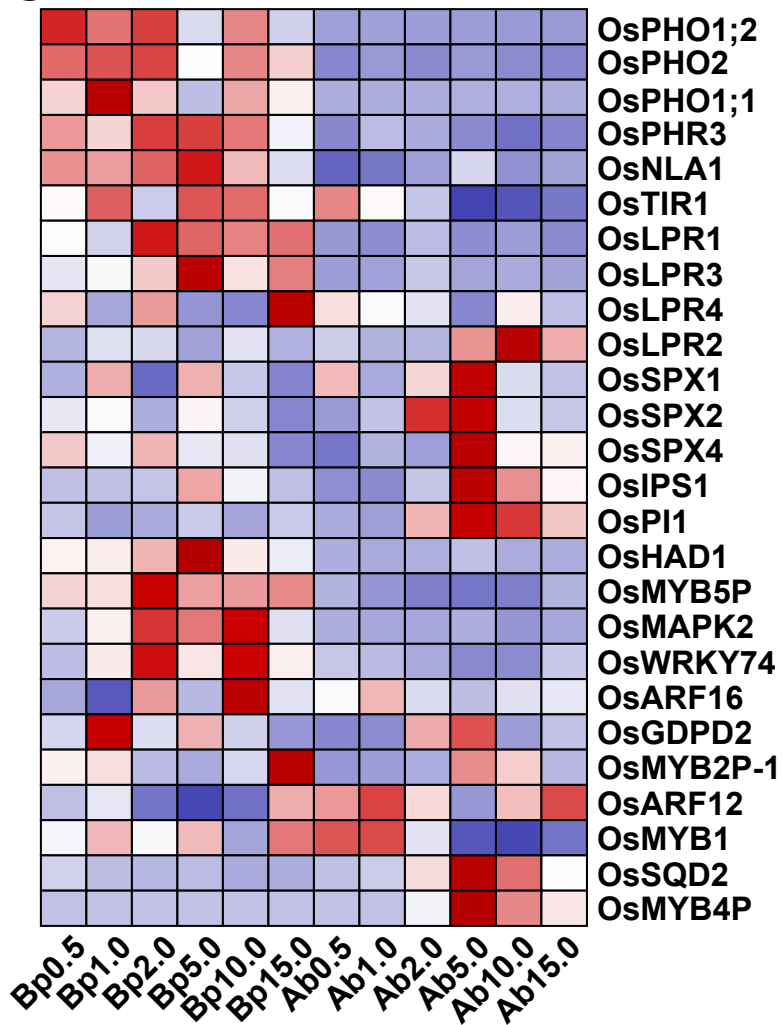

Supplement: Supplementary file 1 — Additional file 1: Table S1. The statistics of reads count of the RNA-seq data. Table S2. The primers used in our study. Table S3. The expression levels of all expressed genes and DEGs. Figure S1. Expression correlation between samples of RNA-seq. (a) Principal component analysis (PCA) shows tissue and N concentration specific samples (with repetition). Ab stands for axillary bud, Bp stands for basal part. (b) Expression correlations between two samples reveals the difference between axillary bud and basal part, and between different N concentrations. _1 and _2 stands for two repetitions. Figure S2. Specific and common genes between basal part and axillary bud under low N and high N conditions. (a) Venn diagram showing the overlaps among responsive genes in basal part and axillary bud under low and high N conditions. (b) The number of total and specific DEGs and TF genes in the four datasets shown in (a). (c) The common gene numbers between axillary bud and basal part under low N and high N conditions. Figure S3. Enriched GO terms within the category of cellular component (a) and molecular function (b) for DEGs in the ten expression patterns (B01-B09) in Fig. 4b. Only significant go terms (false discovery rate (FDR) < 0.05) are displayed. Figure S4. Enriched GO terms within the category of cellular component (a) and molecular function (b) for DEGs in the nine expression patterns (A01-A10) in Fig. 5b. Only significant go terms (false discovery rate (FDR) < 0.05) are displayed. Figure S5. Differentially expressed genes between basal part and axillary bud under each N concentration. (a) The number of up and down-regulated DEGs (fold change> 2 and padj< 0.05 by DESeq2) detected in axillary bud compared with basal part under six N concentrations. The number of up-, down- regulated and total DEGs for each N concentrations are shown. (b) Clustering the total DEGs detected between axillary bud and basal part under six N concentrations. FPKM values were scaled per gene acros [file 12870_2020_2409_MOESM1_ESM.zip › Additional file1-Figure S11.pdf]

a

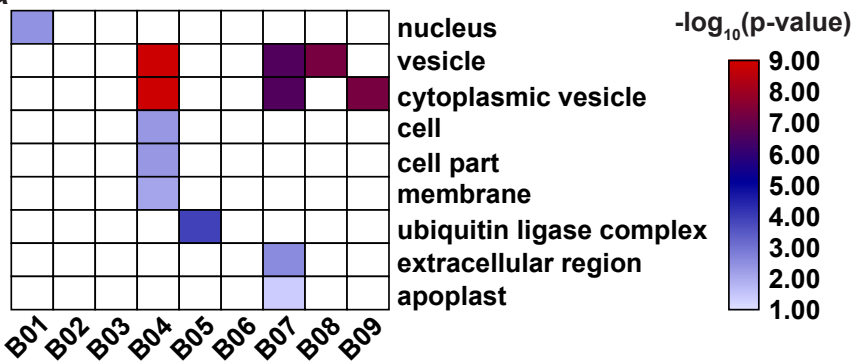

b

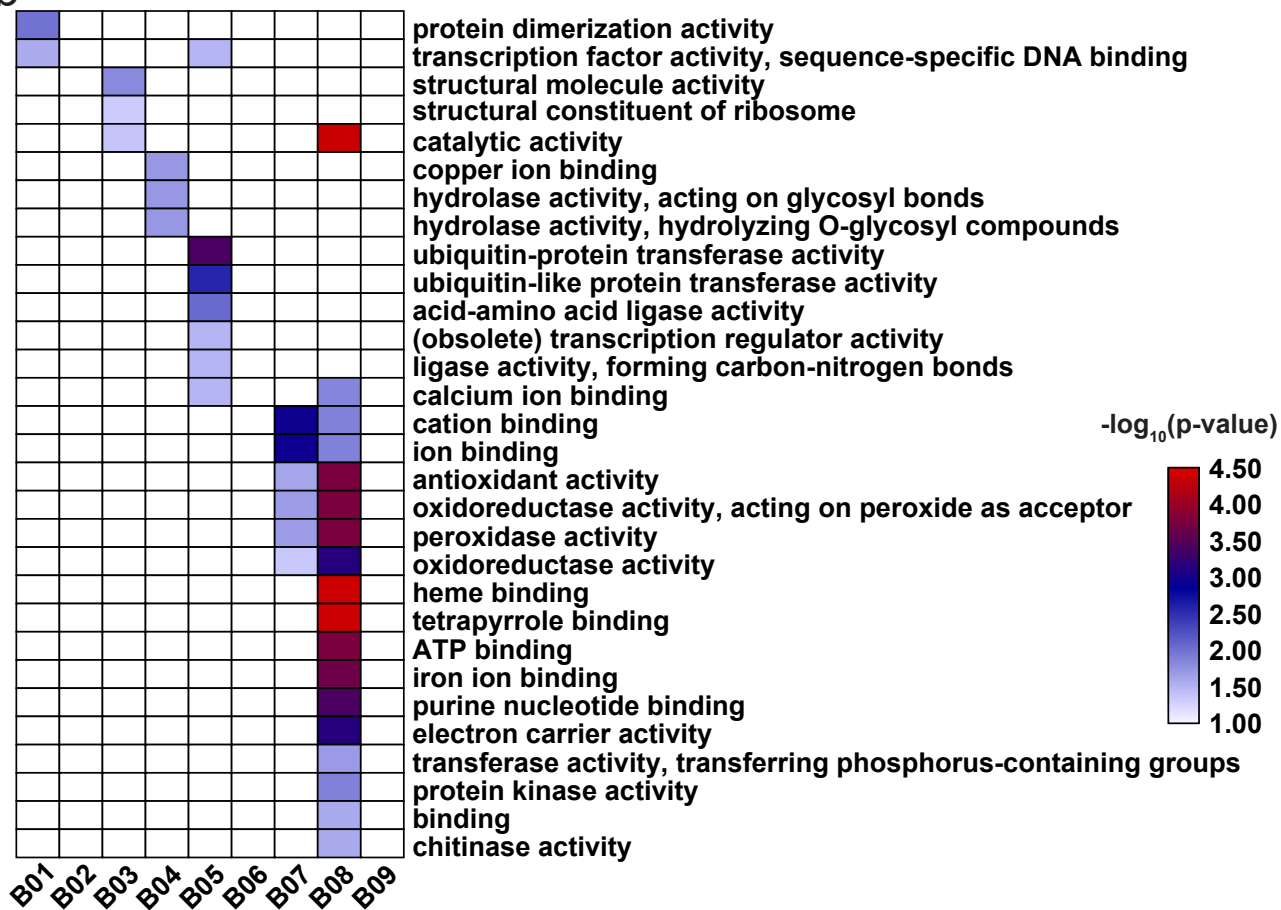

Supplement: Supplementary file 1 — Additional file 1: Table S1. The statistics of reads count of the RNA-seq data. Table S2. The primers used in our study. Table S3. The expression levels of all expressed genes and DEGs. Figure S1. Expression correlation between samples of RNA-seq. (a) Principal component analysis (PCA) shows tissue and N concentration specific samples (with repetition). Ab stands for axillary bud, Bp stands for basal part. (b) Expression correlations between two samples reveals the difference between axillary bud and basal part, and between different N concentrations. _1 and _2 stands for two repetitions. Figure S2. Specific and common genes between basal part and axillary bud under low N and high N conditions. (a) Venn diagram showing the overlaps among responsive genes in basal part and axillary bud under low and high N conditions. (b) The number of total and specific DEGs and TF genes in the four datasets shown in (a). (c) The common gene numbers between axillary bud and basal part under low N and high N conditions. Figure S3. Enriched GO terms within the category of cellular component (a) and molecular function (b) for DEGs in the ten expression patterns (B01-B09) in Fig. 4b. Only significant go terms (false discovery rate (FDR) < 0.05) are displayed. Figure S4. Enriched GO terms within the category of cellular component (a) and molecular function (b) for DEGs in the nine expression patterns (A01-A10) in Fig. 5b. Only significant go terms (false discovery rate (FDR) < 0.05) are displayed. Figure S5. Differentially expressed genes between basal part and axillary bud under each N concentration. (a) The number of up and down-regulated DEGs (fold change> 2 and padj< 0.05 by DESeq2) detected in axillary bud compared with basal part under six N concentrations. The number of up-, down- regulated and total DEGs for each N concentrations are shown. (b) Clustering the total DEGs detected between axillary bud and basal part under six N concentrations. FPKM values were scaled per gene acros [file 12870_2020_2409_MOESM1_ESM.zip › Additional file1-Figure S3.pdf]

**a**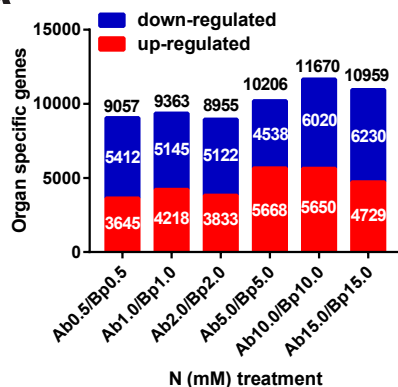**b**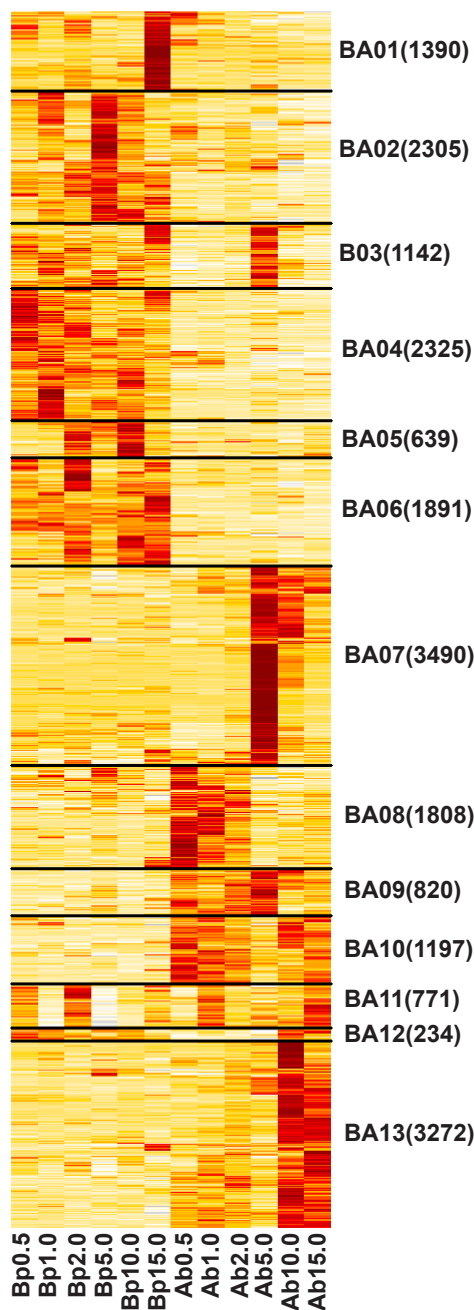**c**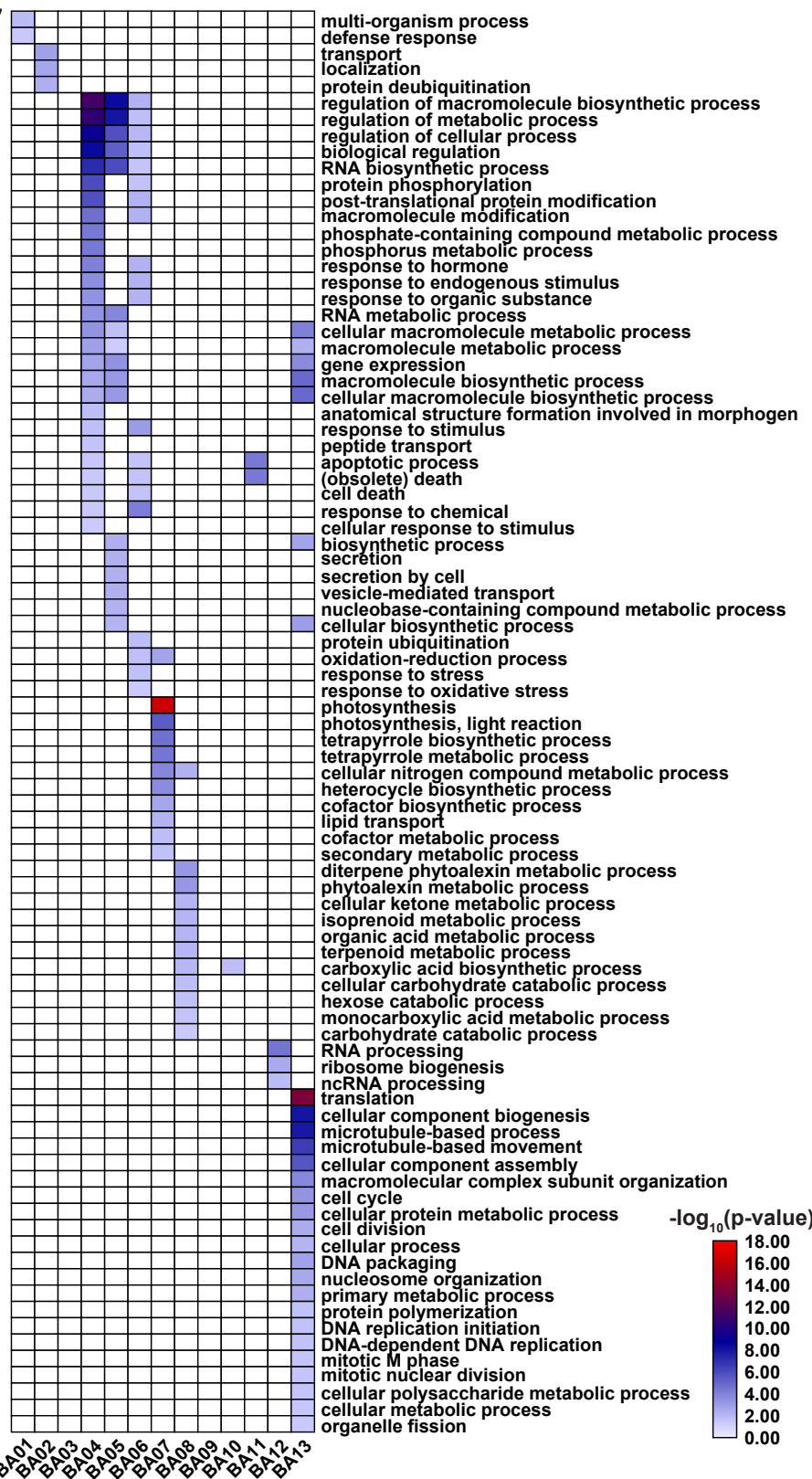

Supplement: Supplementary file 1 — Additional file 1: Table S1. The statistics of reads count of the RNA-seq data. Table S2. The primers used in our study. Table S3. The expression levels of all expressed genes and DEGs. Figure S1. Expression correlation between samples of RNA-seq. (a) Principal component analysis (PCA) shows tissue and N concentration specific samples (with repetition). Ab stands for axillary bud, Bp stands for basal part. (b) Expression correlations between two samples reveals the difference between axillary bud and basal part, and between different N concentrations. _1 and _2 stands for two repetitions. Figure S2. Specific and common genes between basal part and axillary bud under low N and high N conditions. (a) Venn diagram showing the overlaps among responsive genes in basal part and axillary bud under low and high N conditions. (b) The number of total and specific DEGs and TF genes in the four datasets shown in (a). (c) The common gene numbers between axillary bud and basal part under low N and high N conditions. Figure S3. Enriched GO terms within the category of cellular component (a) and molecular function (b) for DEGs in the ten expression patterns (B01-B09) in Fig. 4b. Only significant go terms (false discovery rate (FDR) < 0.05) are displayed. Figure S4. Enriched GO terms within the category of cellular component (a) and molecular function (b) for DEGs in the nine expression patterns (A01-A10) in Fig. 5b. Only significant go terms (false discovery rate (FDR) < 0.05) are displayed. Figure S5. Differentially expressed genes between basal part and axillary bud under each N concentration. (a) The number of up and down-regulated DEGs (fold change> 2 and padj< 0.05 by DESeq2) detected in axillary bud compared with basal part under six N concentrations. The number of up-, down- regulated and total DEGs for each N concentrations are shown. (b) Clustering the total DEGs detected between axillary bud and basal part under six N concentrations. FPKM values were scaled per gene acros [file 12870_2020_2409_MOESM1_ESM.zip › Additional file1-Figure S5.pdf]

a

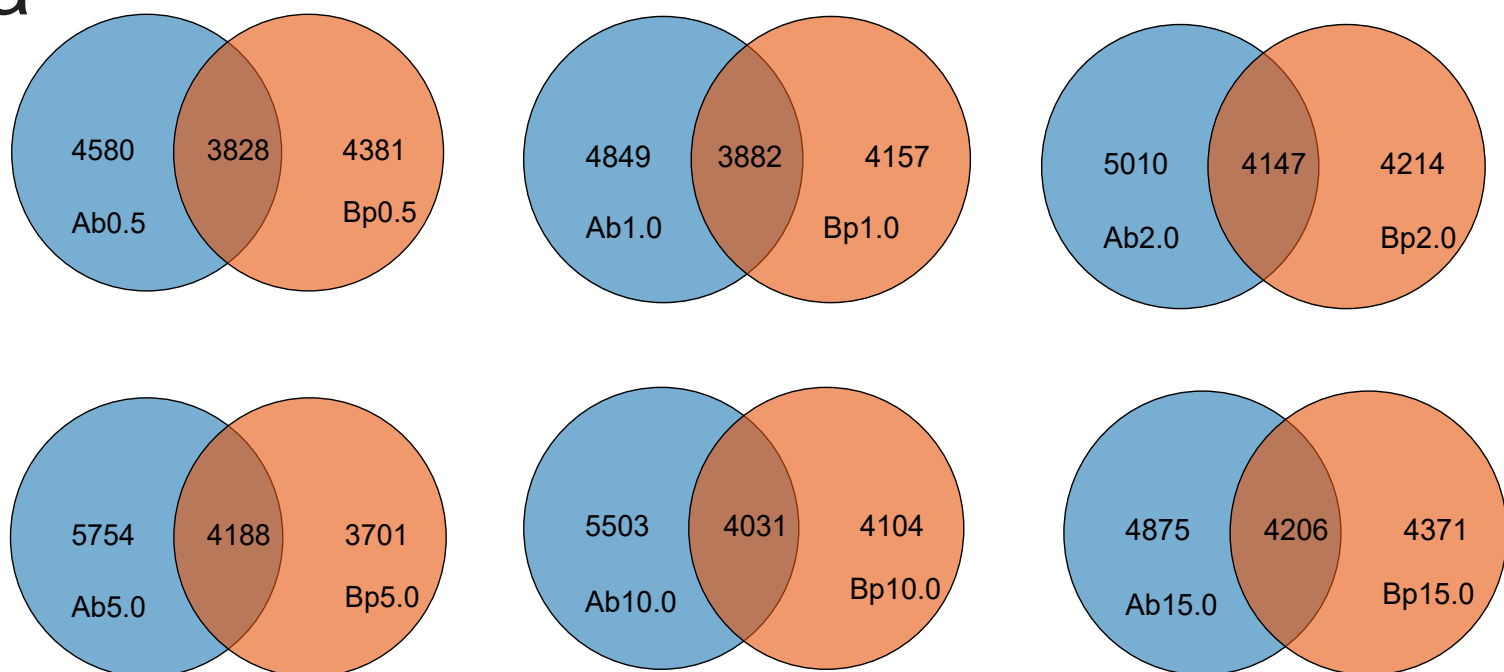

b

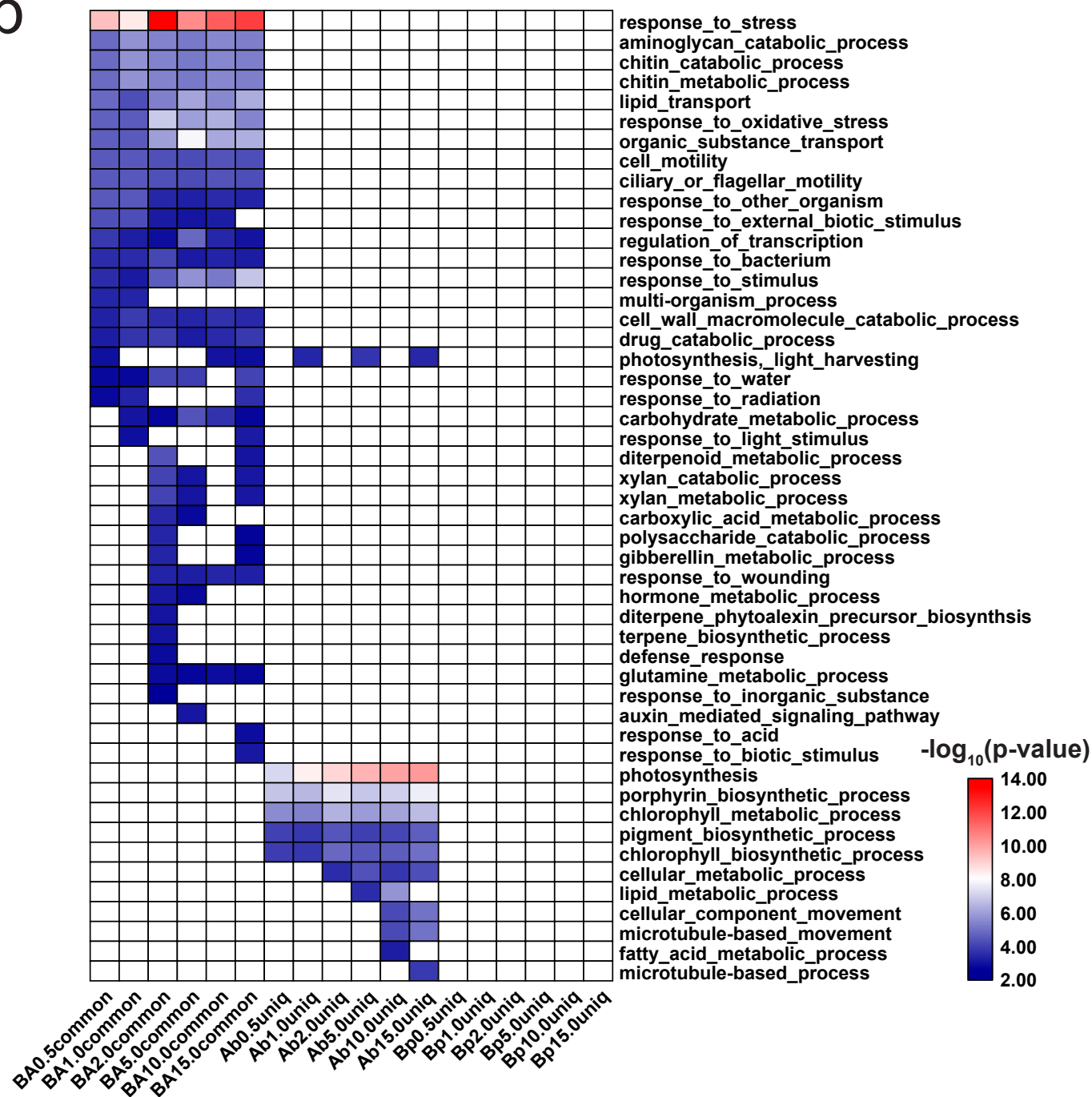

Supplement: Supplementary file 1 — Additional file 1: Table S1. The statistics of reads count of the RNA-seq data. Table S2. The primers used in our study. Table S3. The expression levels of all expressed genes and DEGs. Figure S1. Expression correlation between samples of RNA-seq. (a) Principal component analysis (PCA) shows tissue and N concentration specific samples (with repetition). Ab stands for axillary bud, Bp stands for basal part. (b) Expression correlations between two samples reveals the difference between axillary bud and basal part, and between different N concentrations. _1 and _2 stands for two repetitions. Figure S2. Specific and common genes between basal part and axillary bud under low N and high N conditions. (a) Venn diagram showing the overlaps among responsive genes in basal part and axillary bud under low and high N conditions. (b) The number of total and specific DEGs and TF genes in the four datasets shown in (a). (c) The common gene numbers between axillary bud and basal part under low N and high N conditions. Figure S3. Enriched GO terms within the category of cellular component (a) and molecular function (b) for DEGs in the ten expression patterns (B01-B09) in Fig. 4b. Only significant go terms (false discovery rate (FDR) < 0.05) are displayed. Figure S4. Enriched GO terms within the category of cellular component (a) and molecular function (b) for DEGs in the nine expression patterns (A01-A10) in Fig. 5b. Only significant go terms (false discovery rate (FDR) < 0.05) are displayed. Figure S5. Differentially expressed genes between basal part and axillary bud under each N concentration. (a) The number of up and down-regulated DEGs (fold change> 2 and padj< 0.05 by DESeq2) detected in axillary bud compared with basal part under six N concentrations. The number of up-, down- regulated and total DEGs for each N concentrations are shown. (b) Clustering the total DEGs detected between axillary bud and basal part under six N concentrations. FPKM values were scaled per gene acros [file 12870_2020_2409_MOESM1_ESM.zip › Additional file1-Figure S7.pdf]

**a**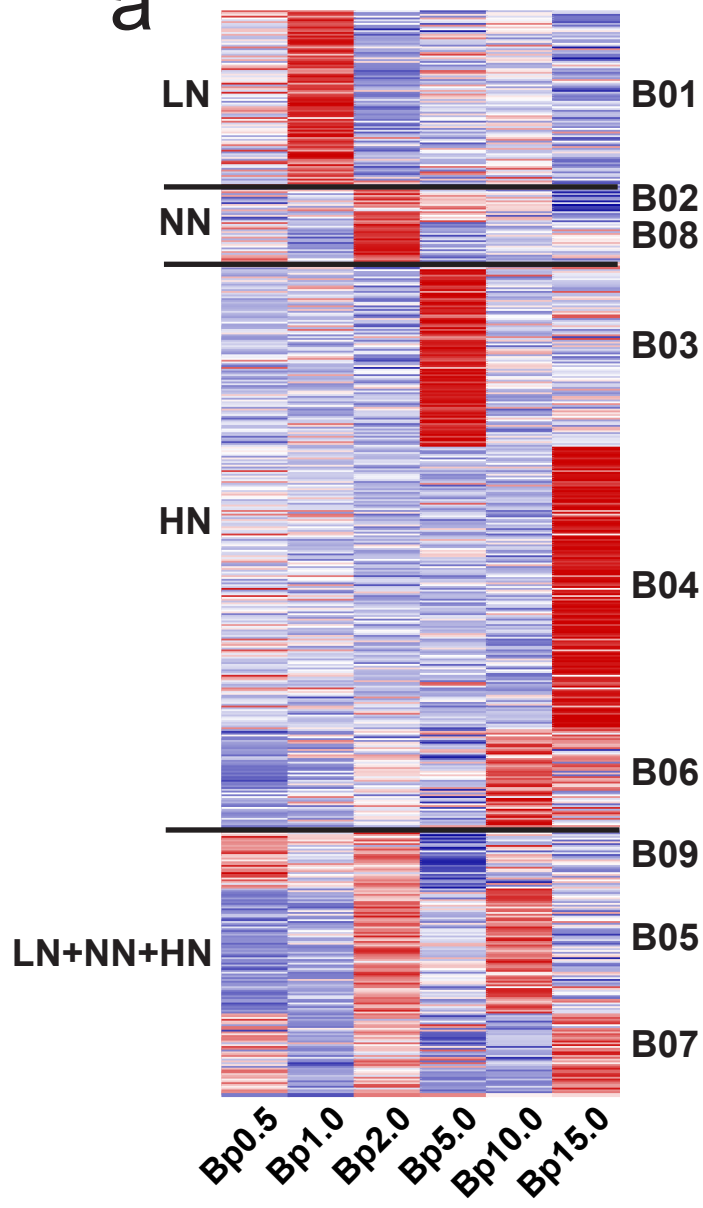**b**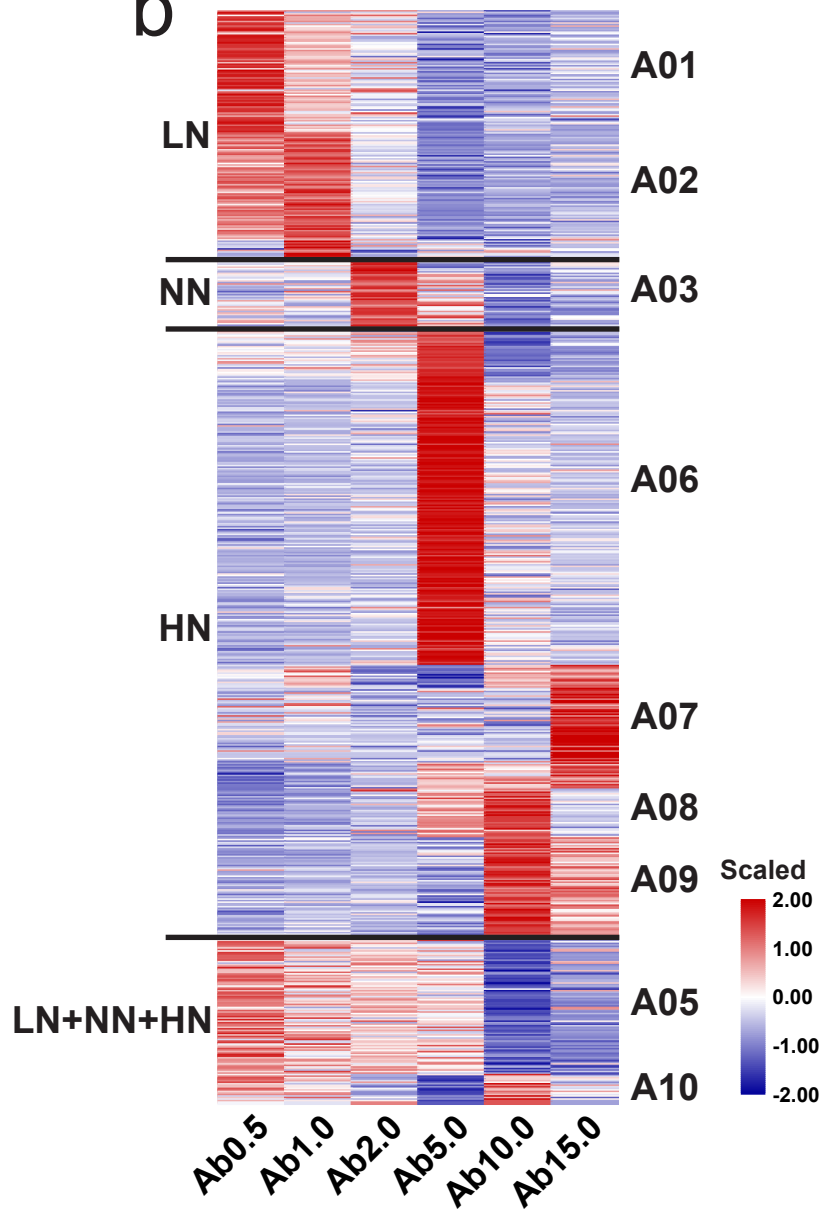

Supplement: Supplementary file 1 — Additional file 1: Table S1. The statistics of reads count of the RNA-seq data. Table S2. The primers used in our study. Table S3. The expression levels of all expressed genes and DEGs. Figure S1. Expression correlation between samples of RNA-seq. (a) Principal component analysis (PCA) shows tissue and N concentration specific samples (with repetition). Ab stands for axillary bud, Bp stands for basal part. (b) Expression correlations between two samples reveals the difference between axillary bud and basal part, and between different N concentrations. _1 and _2 stands for two repetitions. Figure S2. Specific and common genes between basal part and axillary bud under low N and high N conditions. (a) Venn diagram showing the overlaps among responsive genes in basal part and axillary bud under low and high N conditions. (b) The number of total and specific DEGs and TF genes in the four datasets shown in (a). (c) The common gene numbers between axillary bud and basal part under low N and high N conditions. Figure S3. Enriched GO terms within the category of cellular component (a) and molecular function (b) for DEGs in the ten expression patterns (B01-B09) in Fig. 4b. Only significant go terms (false discovery rate (FDR) < 0.05) are displayed. Figure S4. Enriched GO terms within the category of cellular component (a) and molecular function (b) for DEGs in the nine expression patterns (A01-A10) in Fig. 5b. Only significant go terms (false discovery rate (FDR) < 0.05) are displayed. Figure S5. Differentially expressed genes between basal part and axillary bud under each N concentration. (a) The number of up and down-regulated DEGs (fold change> 2 and padj< 0.05 by DESeq2) detected in axillary bud compared with basal part under six N concentrations. The number of up-, down- regulated and total DEGs for each N concentrations are shown. (b) Clustering the total DEGs detected between axillary bud and basal part under six N concentrations. FPKM values were scaled per gene acros [file 12870_2020_2409_MOESM1_ESM.zip › Additional file1-Figure S8.pdf]

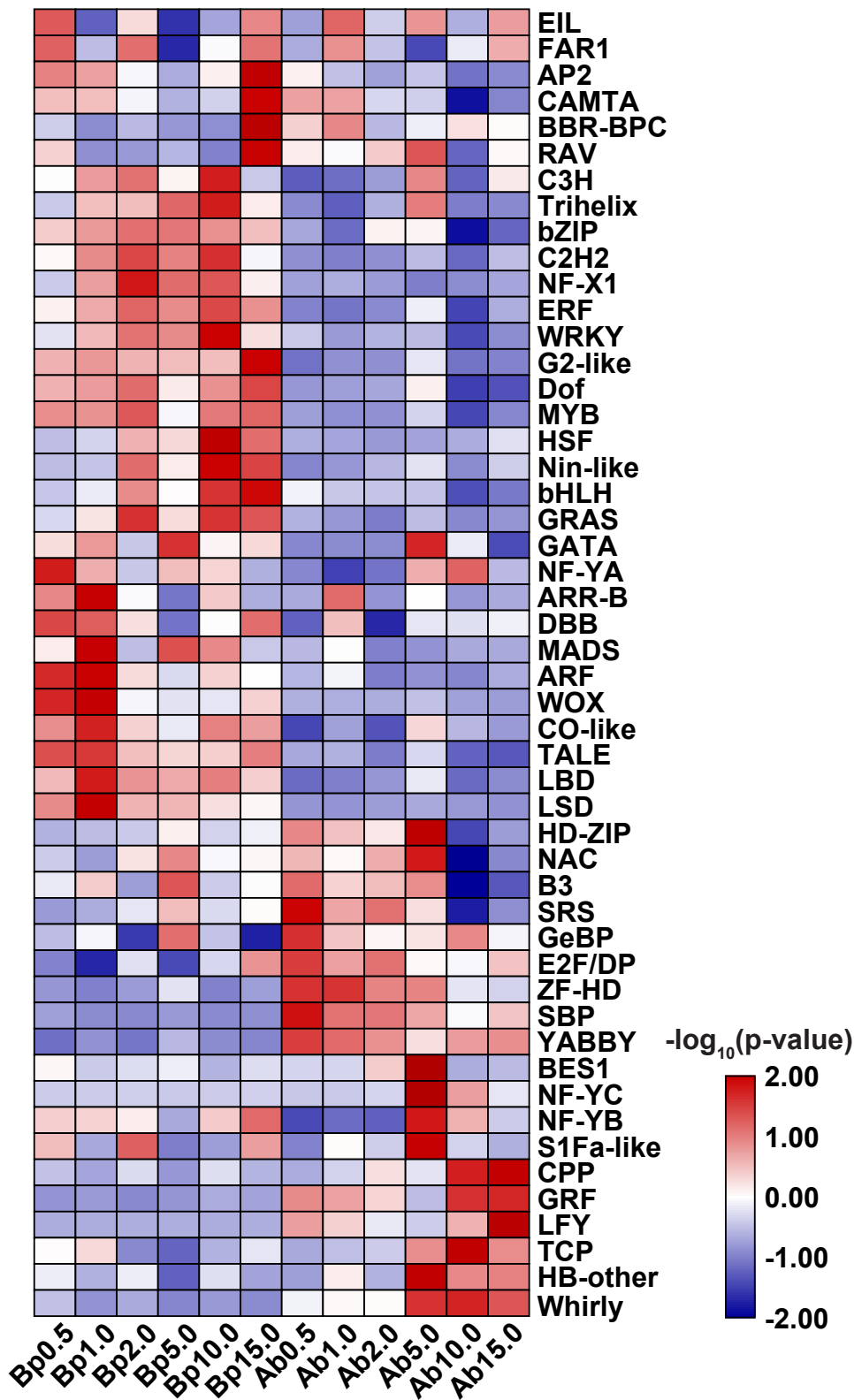

Supplement: Supplementary file 1 — Additional file 1: Table S1. The statistics of reads count of the RNA-seq data. Table S2. The primers used in our study. Table S3. The expression levels of all expressed genes and DEGs. Figure S1. Expression correlation between samples of RNA-seq. (a) Principal component analysis (PCA) shows tissue and N concentration specific samples (with repetition). Ab stands for axillary bud, Bp stands for basal part. (b) Expression correlations between two samples reveals the difference between axillary bud and basal part, and between different N concentrations. _1 and _2 stands for two repetitions. Figure S2. Specific and common genes between basal part and axillary bud under low N and high N conditions. (a) Venn diagram showing the overlaps among responsive genes in basal part and axillary bud under low and high N conditions. (b) The number of total and specific DEGs and TF genes in the four datasets shown in (a). (c) The common gene numbers between axillary bud and basal part under low N and high N conditions. Figure S3. Enriched GO terms within the category of cellular component (a) and molecular function (b) for DEGs in the ten expression patterns (B01-B09) in Fig. 4b. Only significant go terms (false discovery rate (FDR) < 0.05) are displayed. Figure S4. Enriched GO terms within the category of cellular component (a) and molecular function (b) for DEGs in the nine expression patterns (A01-A10) in Fig. 5b. Only significant go terms (false discovery rate (FDR) < 0.05) are displayed. Figure S5. Differentially expressed genes between basal part and axillary bud under each N concentration. (a) The number of up and down-regulated DEGs (fold change> 2 and padj< 0.05 by DESeq2) detected in axillary bud compared with basal part under six N concentrations. The number of up-, down- regulated and total DEGs for each N concentrations are shown. (b) Clustering the total DEGs detected between axillary bud and basal part under six N concentrations. FPKM values were scaled per gene acros [file 12870_2020_2409_MOESM1_ESM.zip › Additional file1-Figure S9.pdf]
