# Supplementary material for: Transcriptomic and physiological analyses of rice seedlings under different nitrogen supplies provide insight into the regulation involved in axillary bud outgrowth
Source: BMC Plant Biol. 2020 May 7;20:197. doi: 10.1186/s12870-020-02409-0 (PMC7206722; doi:10.1186/s12870-020-02409-0)
Supplement: Supplementary file 1 — Additional file 1: Table S1. The statistics of reads count of the RNA-seq data. Table S2. The primers used in our study. Table S3. The expression levels of all expressed genes and DEGs. Figure S1. Expression correlation between samples of RNA-seq. (a) Principal component analysis (PCA) shows tissue and N concentration specific samples (with repetition). Ab stands for axillary bud, Bp stands for basal part. (b) Expression correlations between two samples reveals the difference between axillary bud and basal part, and between different N concentrations. _1 and _2 stands for two repetitions. Figure S2. Specific and common genes between basal part and axillary bud under low N and high N conditions. (a) Venn diagram showing the overlaps among responsive genes in basal part and axillary bud under low and high N conditions. (b) The number of total and specific DEGs and TF genes in the four datasets shown in (a). (c) The common gene numbers between axillary bud and basal part under low N and high N conditions. Figure S3. Enriched GO terms within the category of cellular component (a) and molecular function (b) for DEGs in the ten expression patterns (B01-B09) in Fig. 4b. Only significant go terms (false discovery rate (FDR) < 0.05) are displayed. Figure S4. Enriched GO terms within the category of cellular component (a) and molecular function (b) for DEGs in the nine expression patterns (A01-A10) in Fig. 5b. Only significant go terms (false discovery rate (FDR) < 0.05) are displayed. Figure S5. Differentially expressed genes between basal part and axillary bud under each N concentration. (a) The number of up and down-regulated DEGs (fold change> 2 and padj< 0.05 by DESeq2) detected in axillary bud compared with basal part under six N concentrations. The number of up-, down- regulated and total DEGs for each N concentrations are shown. (b) Clustering the total DEGs detected between axillary bud and basal part under six N concentrations. FPKM values were scaled per gene acros [file Additional file1-Figure S6.pdf › Additional file1-Figure S6.pdf]

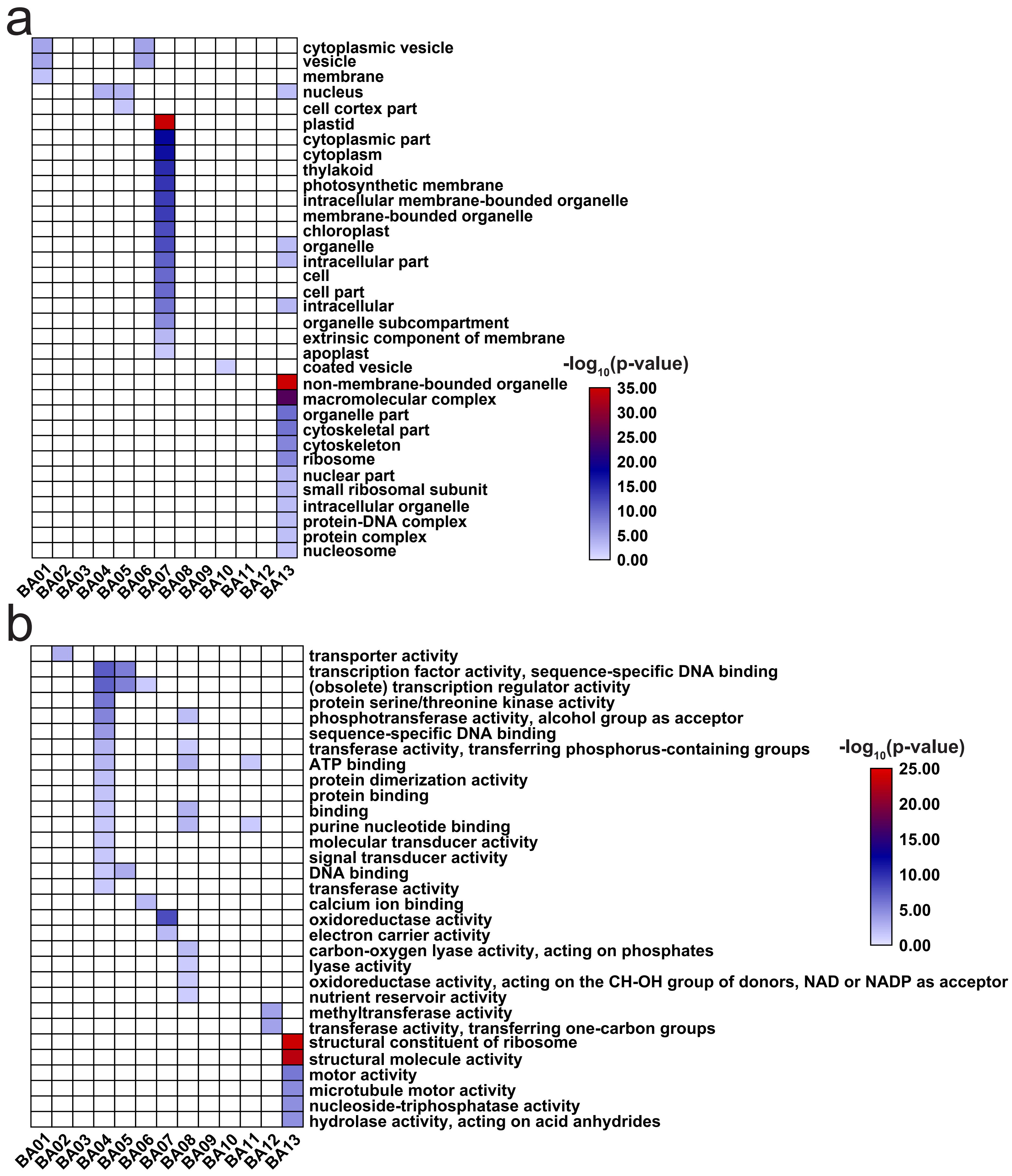
a
cytoplasmic vesicle
vesicle
membrane
nucleus
cell cortex part
plastid
cytoplasmic part
cytoplasm
thylakoid
photosynthetic membrane
intracellular membrane-bounded organelle
membrane-bounded organelle
chloroplast
organelle
intracellular part
cell
cell part
intracellular
organelle subcompartment
extrinsic component of membrane
apoplast
coated vesicle
non-membrane-bounded organelle
macromolecular complex
organelle part
cytoskeletal part
cytoskeleton
ribosome
nuclear part
small ribosomal subunit
intracellular organelle
protein-DNA complex
protein complex
nucleosome
-log10(p-value)
35.00
30.00
25.00
20.00
15.00
10.00
5.00
0.00
BA01 BA02 BA03 BA04 BA05 BA06 BA07 BA08 BA09 BA10 BA11 BA12 BA13
b
transporter activity
transcription factor activity, sequence-specific DNA binding
(obsolete) transcription regulator activity
protein serine/threonine kinase activity
phosphotransferase activity, alcohol group as acceptor
sequence-specific DNA binding
transferase activity, transferring phosphorus-containing groups
ATP binding
protein dimerization activity
protein binding
binding
purine nucleotide binding
molecular transducer activity
signal transducer activity
DNA binding
transferase activity
calcium ion binding
oxidoreductase activity
electron carrier activity
carbon-oxygen lyase activity, acting on phosphates
lyase activity
oxidoreductase activity, acting on the CH-OH group of donors, NAD or NADP as acceptor
nutrient reservoir activity
methyltransferase activity
transferase activity, transferring one-carbon groups
structural constituent of ribosome
structural molecule activity
motor activity
microtubule motor activity
nucleoside-triphosphatase activity
hydrolase activity, acting on acid anhydrides
-log10(p-value)
25.00
20.00
15.00
10.00
5.00
0.00
BA01 BA02 BA03 BA04 BA05 BA06 BA07 BA08 BA09 BA10 BA11 BA12 BA13
